# A genetic study and meta-analysis of the genetic predisposition of prostate cancer in a Chinese population

**DOI:** 10.18632/oncotarget.7250

**Published:** 2016-02-08

**Authors:** Jacek Marzec, Xueying Mao, Meiling Li, Meilin Wang, Ninghan Feng, Xin Gou, Guomin Wang, Zan Sun, Jianfeng Xu, Hua Xu, Xiaoping Zhang, Shan-Chao Zhao, Guoping Ren, Yongwei Yu, Yudong Wu, Ji Wu, Yao Xue, Bo Zhou, Yanling Zhang, Xingxing Xu, Jie Li, Weiyang He, Sara Benlloch, Helen Ross-Adams, Li Chen, Jucong Li, Yingqia Hong, Zsofia Kote-Jarai, Xingang Cui, Jianguo Hou, Jianming Guo, Lei Xu, Changjun Yin, Yuanping Zhou, David E. Neal, Tim Oliver, Guangwen Cao, Zhengdong Zhang, Douglas F. Easton, Claude Chelala, The PRACTICAL Consortium, The CHIPGECS Group, Ali Amin Al Olama, Rosalind A. Eeles, Hongwei Zhang, Yong-Jie Lu

**Affiliations:** ^1^ Centre for Molecular Oncology, Barts Cancer Institute, Barts and the London School of Medicine and Dentistry, Queen Mary University of London, London, EC1M 6BQ, UK; ^2^ Department of Epidemiology, Second Military Medical University, Shanghai, 200433, China; ^3^ Department of Molecular and Genetic Toxicology, The Key Laboratory of Modern Toxicology, School of Public Health, Nanjing Medical University, Nanjing, 210029, China; ^4^ Department of Urology, Wuxi Second People's Hospital, Nanjing Medical University, Wuxi, 214002, China; ^5^ Department of Urology, The First Affiliated Hospital of Nanjing Medical University, Nanjing, 210029, China; ^6^ Department of Urology, The First Affiliated Hospital, Chongqing Medical University, Chongqing, 400016, China; ^7^ Department of Urology, Zhongshan Hospital, Fudan University Medical College, Shanghai, 200032, China; ^8^ Liaoning People's Hospital and Center of Experiment and Technology, China Medical University, Shenyang, 110001, China; ^9^ Program for Personalized Cancer Care, North Shore University Health System, Evanston, IL 60201, U.S.A; ^10^ Fudan Institute of Urology, Huashang Hospital, Fudan University, Shanghai, 200040, China; ^11^ Department of Urology, Tongji Hospital, Huazhong Science and Technology University, Wuhan, 430030, China; ^12^ Department of Urology, Xiehe Hospital, Huazhong Science and Technology University, Wuhan, 430022, China; ^13^ Department of Urology, Nanfang Hospital, Southern Medical University, Guangzhou, 510515, China; ^14^ Department of Pathology, The First Affiliated Hospital, Zhejiang University Medical College, Hangzhou, 310009, China; ^15^ Department of Pathology, Changhai Hospital, The Second Military Medical University, Shanghai, 200433, China; ^16^ Department of Urology, First Affiliated Hospital, Medical College, Zhengzhou University, Zhengzhou, 450003, China; ^17^ Department of Urology, North Sichuan Medical College, Nanchong, 637000, China; ^18^ Department of Nutrition Science, Shenyang Medical College, Shenyang, 110034, China; ^19^ Centre for Cancer Genetic Epidemiology, Department of Public Health and Primary Care, University of Cambridge–Strangeways Research Laboratory, Cambridge, CB1 8RN, UK; ^20^ Cancer Research UK Cambridge Research Institute, Li Ka Shing Centre, Robinson Way, Cambridge, CB2 0RE, UK; ^21^ Division of Genetics and Epidemiology, The Institute of Cancer Research, London, SM2 5NG, UK; ^22^ Department of Urology, Changzheng Hospital, The Second Military Medical University, Shanghai, 200003, China; ^23^ Department of Urology, Changhai Hospital, The Second Military Medical University, Shanghai, 200433, China; ^24^ Department of Infectious Diseases, Nanfang Hospital, Southern Medical University, Guangzhou, 510515, China; ^25^ The Royal Marsden NHS Foundation Trust, London and Surrey, SM2 5NG, UK

**Keywords:** Chinese prostate cancer, genetic risk, single-nucleotide polymorphism, predisposition, population difference

## Abstract

Prostate cancer predisposition has been extensively investigated in European populations, but there have been few studies of other ethnic groups. To investigate prostate cancer susceptibility in the under-investigated Chinese population, we performed single-nucleotide polymorphism (SNP) array analysis on a cohort of Chinese cases and controls and then meta-analysis with data from the existing Chinese prostate cancer genome-wide association study (GWAS). Genotyping 211,155 SNPs in 495 cases and 640 controls of Chinese ancestry identified several new suggestive Chinese prostate cancer predisposition loci. However, none of them reached genome-wide significance level either by meta-analysis or replication study. The meta-analysis with the Chinese GWAS data revealed that four 8q24 loci are the main contributors to Chinese prostate cancer risk and the risk alleles from three of them exist at much higher frequencies in Chinese than European populations. We also found that several predisposition loci reported in Western populations have different effect on Chinese men. Therefore, this first extensive single-nucleotide polymorphism study of Chinese prostate cancer in comparison with European population indicates that four loci on 8q24 contribute to a great risk of prostate cancer in a considerable large proportion of Chinese men. Based on those four loci, the top 10% of the population have six- or two-fold prostate cancer risk compared with men of the bottom 10% or median risk respectively, which may facilitate the design of prostate cancer genetic risk screening and prevention in Chinese men. These findings also provide additional insights into the etiology and pathogenesis of prostate cancer.

## INTRODUCTION

The incidence and mortality rates of prostate cancer vary dramatically among countries with 24-fold and 10-fold difference, respectively, in 2008 [1]. While it is the most common male cancer in most of America, Oceania, western and northern European countries, its incidence in China is much lower. Despite a rapid increase of prostate cancer incidence in China in recent years, the variation between China and Western countries is still large. Both genetic and environmental factors may contribute to this finding, potentially through the induction of different genetic changes [2].

Previous prostate cancer predisposition studies, particularly genome-wide association studies (GWAS), have been mainly performed in populations from European ancestors [3–20]. While certain loci may contribute to prostate cancer in multiple ethnic populations, population differences have been previously demonstrated [3, 16, 18, 21]. Only one GWAS has been performed in the Chinese population [18]. In association with the Prostate Cancer Association Group to Investigate Cancer Associated Alterations in the Genome (PRACTICAL) consortium for prostate cancer predisposition study [6, 8, 15], we investigated genetic predisposition to prostate cancer in men of Han Chinese ancestry using the custom Illumina single-nucleotide polymorphism (SNP) array (iCOGS) [8]. Meta-analysis with the Chinese GWAS data [18] revealed few suggestive loci. Importantly, we found that four loci on 8q24 contribute in a large proportion of Chinese men to a considerable risk of prostate cancer, which are different in the European population.

## RESULTS

### Genotyping and analysis

**Figure 1:**
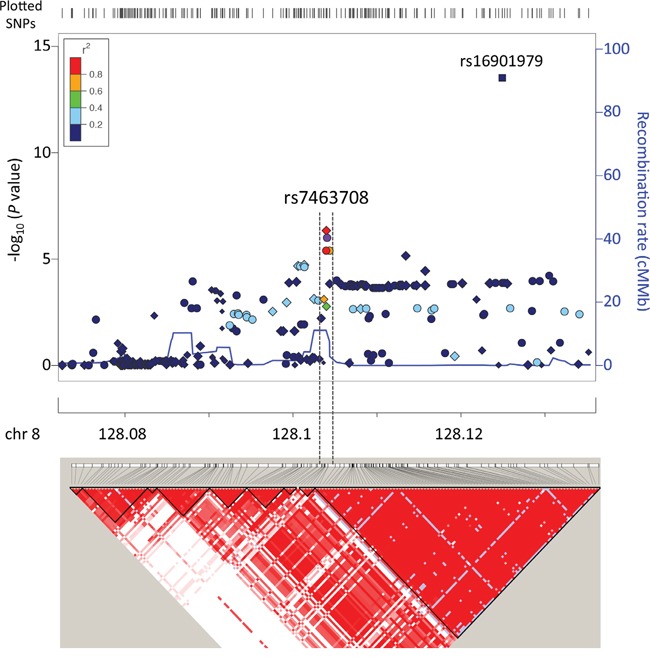
Regional association plot and LD map around SNP rs7463708 Top and bottom panels illustrate regional associations and LD map, respectively, around SNP rs7463708 potentially independently associated with prostate cancer. SNPs genotyped in this study, imputed on the basis of the 1000 Genomes Project data (Phase 1 integrated data version 3, March 2012) and rs16901979 (*P* value based on published data) associated with prostate cancer are indicated by circles, diamonds and square, respectively. Each point size is proportional to sample size. Symbol colors represent the LD of rs7463708 (purple circle) with nearby SNPs. The legend for LD measure (*r*^2^) is on the top left corner of the plot. The left Y-axis illustrates the –log_10_ association *P* values of SNPs and the Y-axis on the right shows the recombination rate estimated from the 1000 Genomes Project using Chinese and Japanese population data. The LD map is based on *r*^2^ values in CHB (Han Chinese in Beijing, China) and JPT (Japanese in Tokyo, Japan) samples from the 1000 Genomes Project.

We genotyped 495 prostate cancer cases and 640 age matched non-cancer male controls (Supplementary Table S1) using the iCOGS SNP array [8]. No population outliers were identified in population structure analysis (Supplementary Figure S1) and 170,264 SNPs in 455 cases and 614 controls were retained for association analysis after systematic quality control. The quantile-quantile plot based on the association results showed minimal evidence of inflation (λ=1.04)(Supplementary Figure S2). We found eight SNPs independently associated with prostate cancer with *P*<1×10^−4^ and *r*^2^<0.5 for linkage disequilibrium (LD) (Table 1). Five of them have not been reported as associated with prostate cancer risk (Supplementary Figure S3) and the other three, all located at 8q24 regions, were previously reported (Table 1). However, the haplotype pattern observed in our genotype data suggests that SNP rs7463708, which is located within the previously reported 8q24 predisposition loci region 2, is potentially independent from region 2 for prostate cancer predisposition in Chinese population (Figure 1) and demonstrates the strongest association with prostate cancer risk at the array stage. For additionally exploratory purposes, we performed a stepwise logistic regression analysis using the three 8q24 SNPs (*P*<1×10^−4^ and *r*^2^<0.5 for LD) and all previously reported prostate cancer predisposition loci located in 8q24 region. The multivariate analysis performed using the stepwise-selected model showed that rs7463708 exhibits independent association with Chinese prostate cancer (Supplementary Table S2). This adds further evidence that rs7463708 represents loci associated with prostate cancer in Chinese population independent from other 8q24 predisposition SNPs.

**Table 1:** SNPs independently associated with prostate cancer risk with *P*<1×10^−4^ at the array genotyping stage of Chinese samples

SNP	Location^a^	Alleles^b^	Study	MAF		OR (95% CI)^c^	*P*_GC_^d^	Previously reported
				Case	Control			
rs12567052	1q32.1 (200494416)	G/A	Chinese^e^	0.241	0.321	0.67 (0.55-0.81)	5.89×10^−4^	No
		G/A	European^f^	0.404	0.399	1.02 (1.00-1.05)	1.69×10^−2^	
rs10235505	7p21.3 (7441152)	G/A	Chinese^e^	0.347	0.266	1.45 (1.20-1.74)	9.09×10^−5^	No
		G/A	European^f^	0.247	0.244	1.02 (0.99-1.05)	2.30×10^−1^	
rs1532980	8p23.1 (9848617)	T/C	Chinese^e^	0.428	0.486	0.70 (0.59-0.84)	8.65×10^−5^	No
		T/C	European^f^	0.252	0.248	1.02 (0.99-1.05)	2.42×10^−1^	
rs7463708	8q24.21 (128104055)	T/G	Chinese^e^	0.232	0.331	0.60 (0.49-0.74)	7.00×10^−7^	?
		G/T	European^f^	0.280	0.260	1.11 (1.07-1.14)	6.87×10^−10^	
rs7013255	8q24.21 (128130487)	T/G	Chinese^e^	0.340	0.257	1.46 (1.21-1.76)	5.90×10^−5^	Yes
		T/G	European^f^	0.053	0.032	1.69 (1.58-1.81)	4.17×10^−48^	
rs13255059	8q24.21 (128530616)	G/A	Chinese^e^	0.206	0.130	1.74 (1.37-2.20)	4.22×10^−6^	Yes
		G/A	European^f^	0.150	0.109	1.45 (1.39-1.50)	5.24×10^−69^	
rs10746597	9q21.31 (82344077)	A/G	Chinese^e^	0.354	0.271	1.52 (1.25-1.85)	2.58×10^−05^	No
		A/G	European^f^	0.415	0.416	0.99 (0.97-1.02)	7.04×10^−1^	
rs1335214	9q33.2 (122847182)	G/C	Chinese^e^	0.467	0.376	1.46 (1.22-1.74)	2.82×10^−5^	No
		C/G	European^f^	0.420	0.421	0.99 (0.97-1.02)	8.16×10^−1^	

Abbreviations: MAF, minor allele frequency.

a Chromosomal and physical (in bracket) locations based on NCBI Human Genome Build 37.

b Major/minor allele.

c Allelic OR with 95% CI for the minor allele in association with prostate cancer risk.

d *P*_GC_ values in Chinese study indicate λ-corrected *P* values.

e iCOGS study on Chinese population.

f PRACTICAL iCOGS study on European population.

### Population genetic risk similarities and differences

To explore the population genetic risk differences we checked our data for previously reported prostate cancer predisposition loci in European and Japanese populations. We found suggestive association (*P*<0.05) in only nine of the 91 (10%) prostate cancer risk SNPs previously identified in European descendants and successfully measured by iCOGS array or imputed. Three of them are located at 8q24 (Supplementary Table S3). Whereas three of the seven (43%) predisposition SNPs initially identified in Japanese population showed association with prostate cancer (*P*<0.05) in our data (Supplementary Table S3 and Supplementary Figure S4). We further estimated the heterogeneity for these predisposition loci between European and Chinese descendants using our Chinese and the PRACTICAL European array data. 24 of 96 showed opposite odds ratio (OR) direction and seven loci demonstrated significant differences (*P*_het_<0.05) in prostate cancer association between Chinese and European data sets (Supplementary Table S3 and Supplementary Figure S5). For the remaining previously reported prostate cancer predisposition loci, no evidence for significant difference between those two populations was found.

### Chinese prostate cancer predisposition meta-analysis

**Figure 2:**
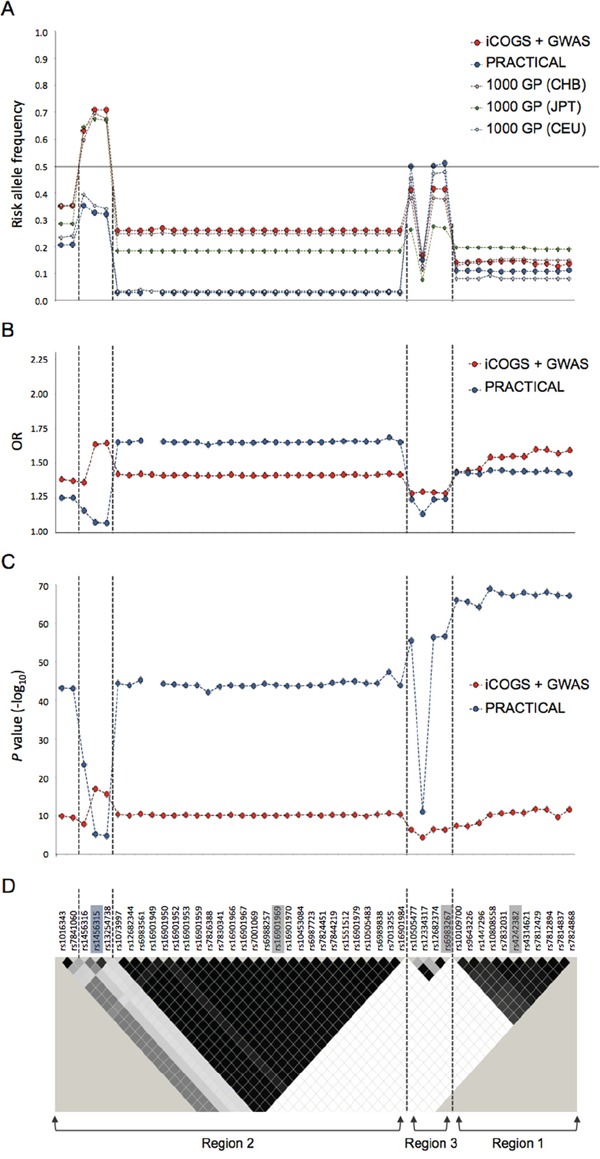
The risk allele frequency, OR and *P* value plots at the four 8q24 prostate cancer-associated loci in Chinese, Japanese and European descendants **A.** Risk allele frequency, **B.** OR and **C.** *P* value plots of SNPs with *P*<1×10^−4^ in meta-analysis results of Chinese data. **D.** LD map of 8q24 region based on *r^2^* values computed using iCOGS Chinese genotyping data. Index SNPs are highlighted in grey on the LD plot. The independently associated SNP rs1456315 in Chinese iCOGS data is highlighted in blue. Previously reported prostate cancer-associated regions 1, 2 and 3 are indicated below the LD map. The vertical dashed lines distinguish the four 8q24 loci independently associated with Chinese prostate cancer accordingly to Chinese iCOGS data. iCOGS: iCOGS array data from Chinese ancestors; PRACTICAL: iCOGS array data from European ancestors; 1000 GP: 1000 Genome Project data; GWAS: Chinese GWAS data.

To increase the statistical power and reduce potential false-positive findings we performed meta-analysis with the Chinese GWAS data [18] using SNPs associated with prostate cancer at *P*<0.001 in our iCOGS array data (Supplementary Table S4). After combining the results from both studies using fixed-effect model, nine loci reached an association with prostate cancer at *P*<1×10^−3^ (Table 2 and Supplementary Table S4). Two previously reported loci at 8q24 (region 1 and 2) reached the GWAS significance (*P*<5×10^−8^) and one (region 3) reached association significance of *P*=2.5×10^−7^. In addition, SNP rs1456315 closely linked with rs7463708 (*r*^2^=0.83), which is absent in Chinese GWAS data, demonstrated the strongest evidence of prostate cancer association in Chinese men (Table 2 and Supplementary Table S4). This is consistent with the Chinese GWAS study identifying rs1456315 as the only SNP with a genome-wide significance level at the GWAS stage [18]. In this Chinese and a previous Japanese study [16], rs1456315 was considered to be within the same LD as the previously reported 8q24 region 2 identified in populations of European ancestry. However, the weak linkage (*r*^2^=0.14) between rs1456315 and region 2 prostate cancer risk susceptibility locus (rs16901979) (Figure 1 and 2D), together with strong association of rs1456315 (OR=0.66, 95% confidence intervals (CI)=0.52-0.82, *P*=2.81×10^−4^) remaining after conditioning on the effects of all the other predisposition loci at 8q24 region using iCOGS array data, suggests that these two loci are independently associated with Chinese prostate cancer. Apart from SNPs at 8q24 region, no other loci were associated with prostate cancer with *P*<1×10^−4^ (Table 2 and Supplementary Table S4).

**Table 2:** Results summary of prostate cancer susceptibility loci with *P*<1×10^−3^ in meta-analysis of Chinese iCOGS and Chinese GWAS data

SNP^a^	Location	Alleles^b^	Reported loci	Study	OR (95% CI)^c^	*P*
rs4539815	2q12	T/C	No	iCOGS	1.42 (1.17-1.74)	5.50×10^−4^
				GWAS	1.14 (1.00-1.31)	6.45×10^−2^
				Combined	1.22 (1.09-1.37)	4.43×10^−4^
rs12622816	2q31	G/A	rs12621278	iCOGS	0.72 (0.59-0.87)	8.92×10^−4^
				GWAS	0.86 (0.76-0.98)	1.94×10^−2^
				Combined	0.82 (0.73-0.91)	1.56×10^−4^
rs13319291	3q22	G/A	No	iCOGS	0.71 (0.59-0.84)	1.37×10^−4^
				GWAS	0.90 (0.80-1.02)	8.40×10^−2^
				Combined	0.83 (0.76-0.92)	3.64×10^−4^
rs1456315	8q24 (region 2)	A/G	No	iCOGS	0.61 (0.50-0.75)	3.78×10^−6^
				GWAS	0.61 (0.53-0.70)	2.76×10^−4^
				Combined	0.61 (0.55-0.68)	7.93×10^−4^
rs7013255	8q24 (region 2)	A/G	rs16901979	iCOGS	1.46 (1.22-1.76)	5.90×10^−5^
				GWAS	1.41 (1.24-1.60)	1.07×10^−7^
				Combined	1.43 (1.29-1.58)	2.28×10^−4^
rs12682374	8q24 (region 3)	G/C	rs6983267	iCOGS	1.36 (1.14-1.61)	5.56×10^−4^
				GWAS	1.26 (1.12-1.42)	1.01×10^−4^
				Combined	1.29 (1.17-1.42)	2.49×10^−7^
rs7824868	8q24 (region 1)	C/T	rs4242382	iCOGS	1.73 (1.37-2.18)	4.61×10^−6^
				GWAS	1.54 (1.32-1.80)	5.14×10^−4^
				Combined	1.60 (1.40-1.82)	2.02×10^−4^
rs6598099	11q13	C/T	No	iCOGS	1.44 (1.18-1.77)	3.97×10^−4^
				GWAS	1.12 (0.98-1.28)	8.53×10^−2^
				Combined	1.21 (1.08-1.35)	8.18×10^−4^
rs1893384	18q11	C/A	No	iCOGS	2.55 (1.50-4.31)	5.20×10^−4^
				GWAS	1.37 (0.94-1.99)	9.95×10^−2^
				Combined	1.69 (1.24-2.29)	7.71×10^−4^

Abbreviation: GWAS, genome-wide association study.

a SNP with the strongest evidence of prostate cancer association in Chinese men accordingly to meta-analysis of Chinese iCOGS and Chinese GWAS data sets.

b Major/minor allele.

c OR with 95% CI for the minor allele in association with prostate cancer risk.

We checked the risk allele frequencies of these 8q24 SNPs in Chinese, Japanese and European populations. Surprisingly, we found that apart from 8q24 region 3, the risk allele frequencies of all SNPs are much higher in Chinese than European population and, for rs1456315 and rs13254738, the risk alleles are the major alleles in Chinese population but minor alleles in European population. The risk allele frequencies for many of these 8q24 SNPs are also higher in Chinese than Japanese population, although the differences are less apparent. Only for some SNPs at 8q24 region 1, the risk allele frequencies are slightly lower in Chinese than Japanese population (Figure 2A). This study also benefits from the direct comparison of the combined Chinese array data with PRACTICAL European population data of 25,074 cases and 24,272 controls. Apart from SNPs between rs1073997 and rs16901984 on 8q24 region 2 (Figure 2D), where the risk allele frequencies are very low (<5%) in European population, all the remaining risk SNPs had higher ORs in Chinese than the European men (Figure 2B). Interestingly, the two SNPs (rs1456315 and rs13254738) demonstrating the highest association with prostate cancer in Chinese population (*P*=7.93×10^−18^ and 2.22×10^−16^ respectively) are not associated with prostate cancer at the GWAS level (*P*<5×10^−8^) in European men (Figure 2C). Due to the high frequency of those risk alleles, the four 8q24 SNPs (rs1456315, rs16901979, rs6983267 and rs4242382) independently associated with Chinese prostate cancer coexist in a considerable large proportion of Chinese men, i.e. 6.5% subjects have six or more of these risk alleles. Based on these four loci, the prostate cancer risk for men in the top 10% of the polygenic risk score (PRS) distribution was 5.91 (95% CI, 2.68-13.04) fold compared with men in the bottom 10%, and around two-fold (1.96, 95% CI, 1.36-2.82) compared with the median risk (Supplementary Table S5). When we looked only at the top 5% of the population, the prostate cancer risk was 8.89 (95% CI, 3.46-22.83) fold compared with men in the bottom 5%, and 2.32 (95% CI, 1.55-3.49) fold compared with the median risk.

### Replication of unreported SNPs

As none of the new suggestive loci (P<0.001) from our iCOGs analysis were supported by the Chinese GWAS data, we attempted to replicate the five association signals passing nominal significance *P*<1×10^−4^ at the iCOGS stage (rs12567052, rs10235505, rs1532980, rs10746597 and rs1335214) using the TaqMan^TM^ method in additional cohort of 1,940 cases and 2,820 controls of Han Chinese ancestry. Association analysis using the same approach as in the iCOGS genotyping stage confirmed two SNPs, rs12567052 at 1q32.1 (*P*=1.9×10^−4^) and rs10235505 at 7p21.3 (*P*=9.9×10^−4^), to have significant association with prostate cancer, while the other three were either not significantly associated (*P*>0.05) or demonstrated opposite effect to that observed at the array stage (Supplementary Table S6). After combining the results from both stages using a meta-analysis assuming fixed-effect, the rs12567052[A] allele at 1q32.1 was associated with an OR of 0.79 for prostate cancer risk (95% CI=0.72-0.86, *P*=2.41×10^−7^) and the rs10235505[A] allele at 7p21.3 was associated with an OR of 1.22 (95% CI=1.12-1.33, *P*=2.4×10^−6^) (Supplementary Table S6). For both SNPs, the allele frequency differed considerably between the populations of European and Chinese (Table 1). In the PRACTICAL iCOGS cohort, which mainly contains European descendants [8], the rs12567052[A] putative protective allele in Chinese population showed significantly higher frequency in cases than controls (*P*=0.017). The rs10235505 did not show association with prostate cancer risk (*P*=0.23) (Table 1).

## DISCUSSION

While more than 100 prostate cancer predisposition loci have been identified [3–20, 22, 23], the majority are from European patients. Identifying the predisposition loci in Asian populations, where prostate cancer incidence is relatively low, may not only help to prevent an increase of incidence rate in these populations, but also contribute to our understanding of the molecular and genetic mechanisms underlying prostate carcinogenesis by evaluating population differences.

In this study we identified a number of suggestive prostate cancer-associated genetic loci in Chinese Han population using the custom iCOGS array. Two of them, rs12567052 and rs10235505, remained to be associated at the replication stage, but none reached the genome-wide level significance when combined all the data. Further studies with much larger sample size are required to determine if rs12567052 and rs10235505 are associated with Chinese prostate cancer risk. The SNP with the strongest association at the array stage was rs7463708 at 8q24 (OR=0.60, 95% CI=0.49-0.74, *P*=7.00×10^−7^), which is consistent with the Chinese GWAS study identifying rs1456315, a SNP in strong linkage with rs7463708, as the only SNP with a genome-wide significance level at the GWAS stage [18]. While this SNP is located within previously reported prostate cancer predisposition loci 8q24 region 2 identified in populations of European ancestry, the haplotype pattern observed in our genotype data and stepwise logistic regression analysis suggest that these two loci are independently associated with prostate cancer in Chinese population.

By comparing our data with prostate cancer predisposition loci reported in European and Japanese populations [4, 6–17, 20, 24–29], we found that many prostate cancer predisposition loci identified in European population are not associated with prostate cancer in Chinese men. Clearly the sample size of our study is relatively small, hence the statistical power is limited and with increased sample size more reported predisposition loci to the European men are likely to be confirmed in the Chinese population. However, the much greater proportion of predisposition loci identified in Japanese population compared to those identified in European populations, which demonstrated an association (*P*<0.05) in our Chinese cohort, suggests that these two Eastern Asian populations have more similar genetic risk to prostate cancer than European descent. Moreover, we observed prominent difference in allele frequency in the 8q24 loci and several loci reported in Western populations that have significantly different effect on Chinese men, demonstrating that certain population difference in prostate cancer predisposition exists. However, while we identified differences between Chinese and European populations for certain previously reported prostate cancer predisposition loci, the majority of them did not demonstrate significant heterogeneity between data sets derived from Chinese and European descendants. This is convergent with the presumption that many genetic predisposition loci may contribute to prostate cancer in multiple ethnic populations.

The current sample size of microarray data for Chinese prostate cancer predisposition study is still limited. Therefore, susceptibility loci with very low penetrance may not be detected due to insufficient statistical power. Further Chinese studies with larger cohort of samples are required to comprehensively characterize the genetic predisposition of prostate cancer in Chinese men. However, the much greater significance of prostate cancer association at these four 8q24 SNPs compared with other SNPs in the Chinese array data sets indicate that other loci potentially to be confirmed or identified in Chinese population may have much smaller contribution to prostate cancer risk in Chinese population. As a large proportion (around 6.5%) of Chinese men carry six or more of these 8q24 risk alleles and the top 10% of the population have six- and two-fold prostate cancer risk compared with men of the bottom 10% and median risk respectively, these four 8q24 SNPs may be implemented in a simple prostate cancer genetic risk screening kit to detect these high-risk Chinese men for individualized cancer prevention. In addition to individual genetic risk assessment for cancer prevention, SNP-based prediction model for prostate cancer diagnosis has also been developed [30, 31]. In a previously reported 24 SNP-based genetic score for prostate cancer diagnosis prediction in Chinese population, three of the four SNPs in our study, rs16901979, rs6983267 and rs1447295, have been genotyped. We compared the performance of our three-SNP combination in comparison to the 24 SNPs and the area under the curve (AUC) for our three SNPs was 0.646 and the AUC for the 24 SNPs was 0.658. Although the 24 SNPs performed slightly better than the three SNPs, the cancer prediction value is similar. It is possible that including the 4th SNP, rs1456315, which demonstrated the strongest signal in the Chinese iCOGS and GWAS, could further improve the AUC, suggesting these four 8q24 SNPs may also be useful for diagnosis prediction. This genetic score data also support our observation that the other SNPs have limited contribution to prostate cancer risk in Chinese men.

While those findings suggest that Chinese population has fewer genetic risk loci for prostate cancer than European population, which is consistent with the prostate cancer incidence disparity between these two populations, it is surprising that the risk alleles are more frequent and ORs are greater at these four 8q24 loci in Chinese than European descendants. Our findings suggest that Chinese population either generally has a lower genetic risk or is better protected by healthy diet than European population, resulting in much lower incidence and mortality of prostate cancer. If the former is true, it means that the recent rapid increase in Chinese prostate cancer incidence will peak soon and won't ever achieve European levels. However, if the latter is the case, with dietary change and increased population, prostate cancer will become a common cancer in China.

In summary, while we did not identify new prostate cancer predisposition loci at genome-wide significance level in Chinese population using our SNP array data, meta-analysis and replication study, the meta-analysis with Chinese GWAS showed that four 8q24 loci increase the risk of prostate cancer considerably in a large proportion of Chinese men. These four 8q24 loci bear great potential for designing a simple prostate cancer risk screening kit to identify risk individuals in the large Chinese population. We also identified differences between Chinese and European populations for certain previously reported prostate cancer predisposition loci. These findings will help to understand the predisposition and molecular mechanisms of prostate carcinogenesis and improve cancer prevention.

## MATERIALS AND METHODS

### Study design

In this study 211,155 SNPs were genotyped in 495 cases and 640 age-matched non-cancer male controls from the Han Chinese population using iCOGS array [8] at Beijing Genome Institute. The array was designed by the international consortium, COGS, to detect genetic variants related to prostate, breast and ovarian cancers. The replication of nominally associated SNPs within unreported loci was performed in additional 1,940 cases and 2,820 controls. Demographic and clinical features of subjects for the prostate cancer predisposition study are summarized in Supplementary Table S1. These male Han Chinese subjects were recruited from several regions of China (south, southeast, southwest, middle and northeast) by members of the Chinese Prostate Cancer Genetic and Environmental Correlation Study (CHIPGECS) collaborative group. The cases were hospital based with pathological diagnosis of primary prostate cancer. Similar numbers of cancer-free controls and cases were recruited from the community or hospital subjects undergoing routine physical examination in each recruitment region in China. Controls are all males with comparable ages to the cases, particularly for the first stage conducted with iCOGS array. Blood samples were obtained with informed consent and the study was approved by the Institutional Ethical Committee of each participating institution. The Chinese GWAS [18] data used for meta-analysis contained 1,417 cases and 1,008 cancer-free male controls from the Han Chinese population. The GWAS study subjects selection and genotype data quality control methods were previously described [18].

### Genotype quality control

Systematic quality control steps were conducted on the raw iCOGS genotyping data resulting in the exclusion of 15 samples with an overall genotyping success rate less than 95%, four samples with high B allele frequency (BAF) variance, ten samples predicted as female based on X chromosome specific inbreeding coefficient (F) less than 0.2, and two samples with low heterozygosity (F>0.1). Additional 35 samples identified as duplicates or second-degree relatives were excluded on the basis of identity-by-descent analysis performed using PLINK (v1.07) [32].

Ancestry and population stratification were determined by principal component analysis. The principal components were calculated for a subset of 66,878 uncorrelated (r^2^<0.5) autosomal SNPs that passed quality control and were present on both the iCOGS array and in HapMap 3 data from four reference populations including CEU (Utah residents with Northern and Western European ancestry), CHB (Han Chinese in Beijing, China), JPT (Japanese in Tokyo, Japan) and YRI (Yoruba in Ibadan, Nigeria)(Supplementary Figure S1A). Subsequently, principal components were calculated for a set of 71,591 uncorrelated autosomal markers present on the iCOGS array alone (Supplementary Figure S1B).

The exclusion criteria for SNPs were genotyping call rate less than 95% (3,174 SNPs), minor allele frequency (MAF) less than 1% (37,920 SNPs) and genotype frequency that deviated from expected Hardy-Weinberg equilibrium among control samples (*P*<1×10^−4^) (1,039 SNPs).

### Statistical analyses

The association between each SNP and the disease phenotype was estimated by per-allele OR and 95% CI using unconditional logistic regression implemented in PLINK, assuming an additive genetic model. A genomic control (GC) method was applied to adjust the *P* values for unknown genetic heterogeneity. Ungenotyped SNPs within 100kb flanking regions on each side of associated SNPs (*P*<1×10^−4^) were imputed with IMPUTE2 (version 2.3.0) software[33] using 1000 Genomes Project data (Phase 1 integrated data version 3, March 2012) from Chinese and Japanese populations as reference haplotype maps. A pre-phasing technique was applied to SNP array data to produce best-guess haplotypes and speed up the imputation process[34]. A posterior probability greater than 0.9 was applied to call imputed genotypes. Haploview software (v4.2)[35] was used to determine pair-wise LD structure across interrogated genomic regions. CLUMP analysis with PLINK was performed to identify SNPs independently associated with the disease phenotype. Akaike information criterion (AIC) was used for stepwise logistic regression model selection. Conditional and stepwise logistic regression analyses were performed in R.

Results obtained in independent genotyping analysis were combined with fixed-effect meta-analysis implemented in PLINK. The heterogeneity between studies was tested with Cochran's Q test statistic. Results that obtained *P* value less than 5.0×10^−8^ were considered statistically significant at the genome-wide level.

The different prostate cancer risk strata were defined by PRS constructed from the summed genotypes weighted by the estimated per-allele log-ORs obtained in meta-analysis for each of the four 8q24 loci (Supplementary Table S7)[36].

## SUPPLEMENTARY FIGURES AND TABLES




