# Supplementary material for: A genetic study and meta-analysis of the genetic predisposition of prostate cancer in a Chinese population
Source: Oncotarget. 2016 Feb 8;7(16):21393–403. doi: 10.18632/oncotarget.7250 (PMC5008293; doi:10.18632/oncotarget.7250)
Supplement: Supplementary file 1 [file oncotarget-07-21393-s001.pdf]

SUPPLEMENTARY DATA

CHIPGECS and PRACTICAL co-authorship list

The CHIPGECS (Chinese Prostate Cancer Genetic and Environmental Correlation Study) Group

A Chinese prostate cancer collaborative study group formed by all the authors from the Chinese institutions in the author list as well as Yong Jie Lu and Xueying Mao from Barts Cancer Institute, Queen Mary University of London.

The PRACTICAL Consortium (http://practical.ccge.medschl.cam.ac.uk/)

Rosalind Eeles [1, 2], Doug Easton [3], Zsofia Kote-Jarai [1], Ali Amin Al Olama [3], Sara Benlloch [3], Kenneth Muir [4], Graham G. Giles [5, 6], Fredrik Wiklund [7], Henrik Gronberg [7], Christopher A. Haiman [8], Johanna Schleutker [9, 10], Maren Weischer [11], Ruth C. Travis [12], David Neal [13], Paul Pharoah [14], Kay-Tee Khaw [15], Janet L. Stanford [16, 17], William J. Blot [18], Stephen Thibodeau [19], Christiane Maier [20, 21], Adam S. Kibel [22, 23], Cezary Cybulski [24], Lisa Cannon-Albright [25], Hermann Brenner [26,27], Jong Park [28], Radka Kaneva [29], Jyotsna Batra [30], Manuel R. Teixeira [31], Hardev Pandha[32]

[1] The Institute of Cancer Research, 15 Cotswold Road, Sutton, Surrey, SM2 5NG, UK, [2]Royal Marsden NHS Foundation Trust, Fulham and Sutton, London and Surrey, UK, [3] Centre for Cancer Genetic Epidemiology, Department of Public Health and Primary Care, University of Cambridge, Strangeways Laboratory, Worts Causeway, Cambridge, UK, [4] University of Warwick, Coventry, UK, [5]Cancer Epidemiology Centre, Cancer Council Victoria, 615 St Kilda Road, Melbourne Victoria, Australia, [6] Centre for Epidemiology and Biostatistics, Melbourne School of Population and Global Health, The University of Melbourne, Victoria, Australia, [7] Department of Medical Epidemiology and Biostatistics, Karolinska Institute, Stockholm, Sweden, [8]Department of Preventive Medicine, Keck School of Medicine, University of Southern California/Norris Comprehensive Cancer Center, Los Angeles, California, USA, [9] Department of Medical Biochemistry and Genetics, University of Turku, Turku, Finland, [10] Institute of Biomedical Technology/BioMediTech, University of Tampere and FimLab Laboratories, Tampere, Finland, [11] Department of Clinical Biochemistry, Herlev Hospital, Copenhagen University Hospital, Herlev Ringvej 75, DK-2730 Herlev, Denmark, [12]Cancer Epidemiology Unit, Nuffield Department of Clinical Medicine, University of Oxford, Oxford, UK, [13] Surgical Oncology (Uro-Oncology: S4), University of Cambridge, Box 279, Addenbrooke's Hospital, Hills Road, Cambridge, UK and Cancer Research UK Cambridge Research Institute, Li Ka Shing Centre, Cambridge, UK, [14] Centre for Cancer Genetic Epidemiology, Department of Oncology, University of Cambridge, Strangeways Laboratory, Worts Causeway, Cambridge, UK, [15] Cambridge Institute of Public Health, University of Cambridge, Forvie Site, Robinson Way, Cambridge CB2 0SR, [16] Division of Public Health Sciences, Fred Hutchinson Cancer Research Center, Seattle, Washington, USA, [17] Department of Epidemiology, School of Public Health, University of Washington, Seattle, Washington, USA, [18] International Epidemiology Institute, 1455 Research Blvd., Suite 550, Rockville, MD 20850, [19] Mayo Clinic, Rochester, Minnesota, USA, [20] Department of Urology, University Hospital Ulm, Germany, [21] Institute of Human Genetics University Hospital Ulm, Germany, [22] Brigham and Women's Hospital/Dana-Farber Cancer Institute, 45 Francis Street- ASB II-3, Boston, MA 02115, [23] Washington University, St Louis, Missouri, [24] International Hereditary Cancer Center, Department of Genetics and Pathology, Pomeranian Medical University, Szczecin, Poland, [25] Division of Genetic Epidemiology, Department of Medicine, University of Utah School of Medicine[26] Division of Clinical Epidemiology and Aging Research & Division of Preventive Oncology, German Cancer Research Center, Heidelberg Germany, [27]German Cancer Consortium (DKTK), German Cancer Research Center (DKFZ), Heidelberg Germany, [28]Division of Cancer Prevention and Control, H. Lee Moffitt Cancer Center, 12902 Magnolia Dr., Tampa, Florida, USA, [29] Molecular Medicine Center and Department of Medical Chemistry and Biochemistry, Medical University - Sofia, 2 Zdrave St, 1431, Sofia, Bulgaria, [30] Australian Prostate Cancer Research Centre-Qld, Institute of Health and Biomedical Innovation and Schools of Life Science and Public Health, Queensland University of Technology, Brisbane, Australia, [31] Department of Genetics, Portuguese Oncology Institute, Porto, Portugal and Biomedical Sciences Institute (ICBAS), Porto University, Porto, Portugal, [32]The University of Surrey, Guildford, Surrey, GU2 7XH, UK

SUPPLEMENTAL ACKNOWLEDGMENTS

PRACTICAL & COGS acknowledgement and funding

This study would not have been possible without the contributions of the following: Per Hall (COGS); Douglas F. Easton, Paul Pharoah, Kyriaki Michailidou, Manjeet K. Bolla, Qin Wang (BCAC), Andrew Berchuck (OCAC), Rosalind A. Eeles, Douglas F. Easton, Ali Amin Al Olama, Zsofia Kote-Jarai, Sara Benlloch (PRACTICAL), Georgia Chenevix-Trench, Antonis Antoniou, Lesley McGuffog, Fergus Couch and Ken Offit (CIMBA), Joe Dennis, Alison M. Dunning, Andrew Lee, and Ed Dicks, Craig Luccarini and the staff of the Centre for Genetic Epidemiology Laboratory, Javier Benitez, Anna Gonzalez-Neira and the staff of the CNIO genotyping unit, Jacques Simard and Daniel C. Tessier, Francois Bacot, Daniel Vincent, Sylvie LaBoissière and Frederic Robidoux and the staff of the McGill University and Génome Québec Innovation Centre, Stig E. Bojesen, Sune F. Nielsen, Borge G. Nordestgaard, and the staff of the Copenhagen DNA laboratory, and Julie M. Cunningham, Sharon A. Windebank, Christopher A. Hilker, Jeffrey Meyer and the staff of Mayo Clinic Genotyping Core Facility

Funding for the iCOGS infrastructure came from: the European Community's Seventh Framework Programme under grant agreement n° 223175 (HEALTH-F2-2009-223175) (COGS), Cancer Research UK (C1287/A10118, C1287/A 10710, C12292/A11174, C1281/A12014, C5047/A8384, C5047/A15007, C5047/A10692, C8197/A16565), the National Institutes of Health (CA128978) and Post-Cancer GWAS initiative (1U19 CA148537, 1U19 CA148065 and 1U19 CA148112 - the GAME-ON initiative), the Department of Defence (W81XWH-10-1-0341), the Canadian Institutes of Health Research (CIHR) for the CIHR Team in Familial Risks of Breast Cancer, Komen Foundation for the Cure, the Breast Cancer Research Foundation, and the Ovarian Cancer Research Fund.

SUPPLEMENTARY FIGURES AND TABLES

A

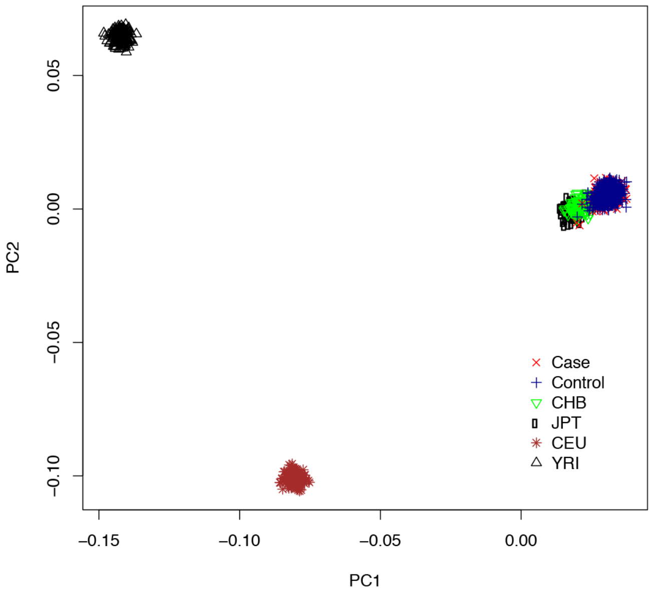

B

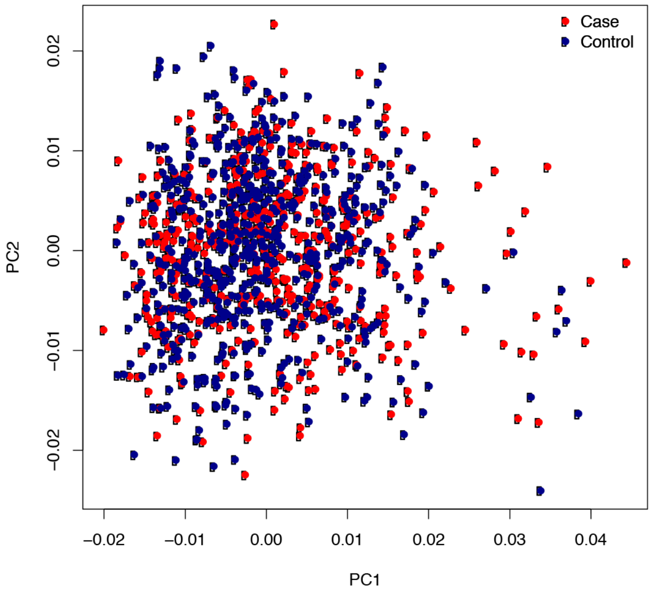

Supplementary Figure S1: Population structure analysis. A. Principal components plot of data from the iCOGS array combined with data derived from Chinese (CHB), Japanese (JPT), European (CEU) and African (YRI) populations in the HapMap3 project based on a subset of 66,878 uncorrelated (r^2<0.5) autosomal SNPs. **B.** Principal components plot of the iCOGS data alone based on a set of 71,591 uncorrelated (r^2<0.5) autosomal SNPs.

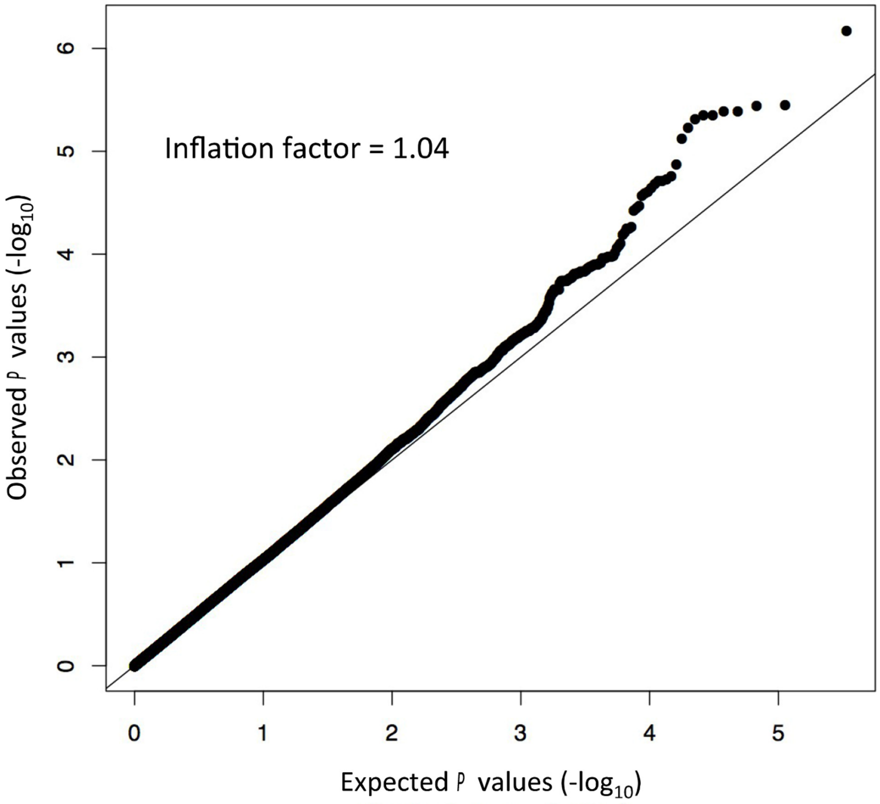

Supplementary Figure S2: Quantile-quantile plot of observed *P* values (-log$_{10}$) plotted as a function of theoretical *P* values for associations with prostate cancer risk calculated using unconditional logistic regression assuming additive genetic model. The solid line indicates where the observed distribution of *P* values is the same as the expected distribution given the number of SNPs tested. The calculated genomic inflation factor (λ) was equal to 1.04.

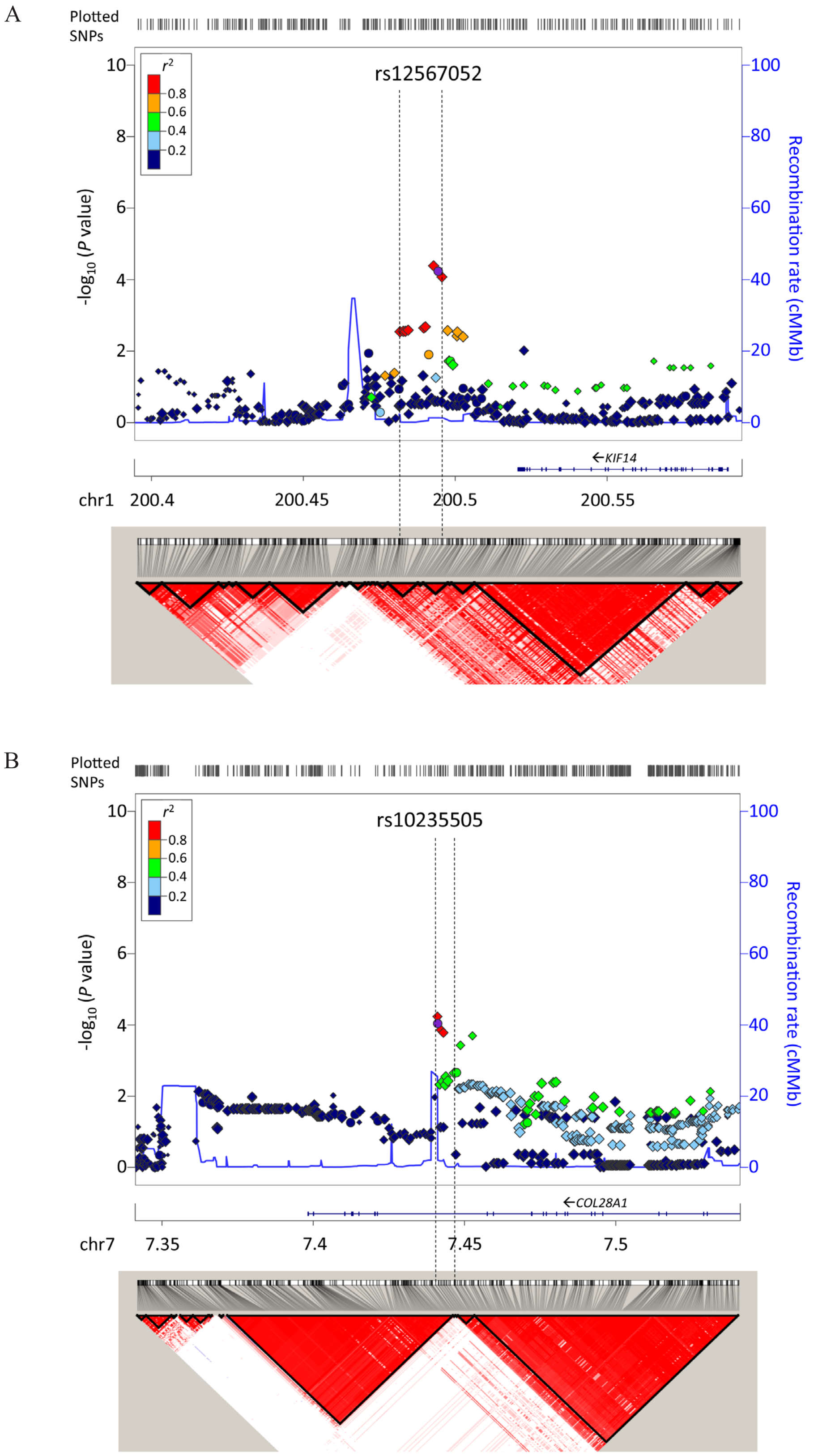

Supplementary Figure S3: Regional association plots and linkage disequilibrium (LD) maps around five new SNPs independently associated with prostate cancer with $P<1\times10^{-4}$ at array stage: A. rs12567052, **B.** rs10235505.

(*Continued*)

C

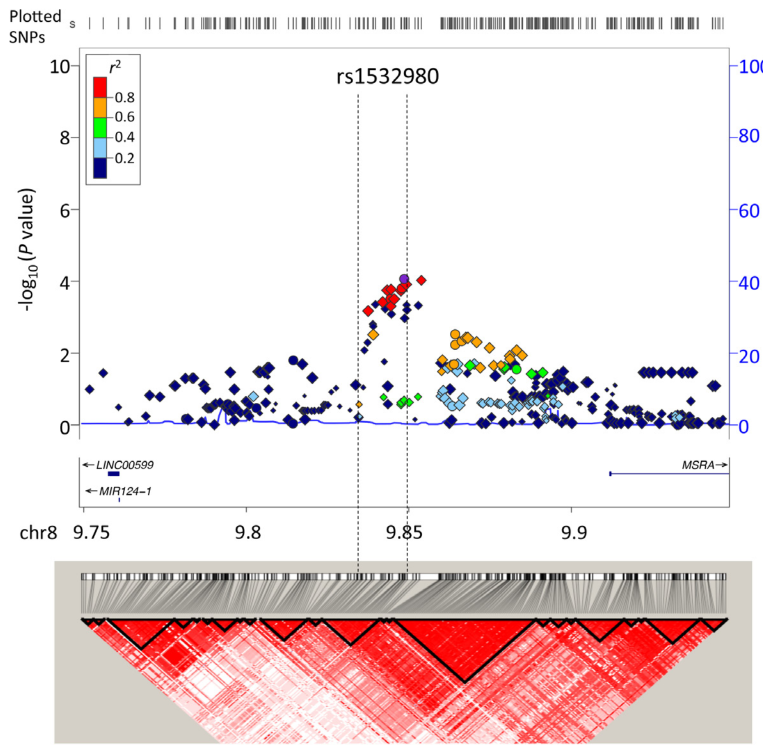

D

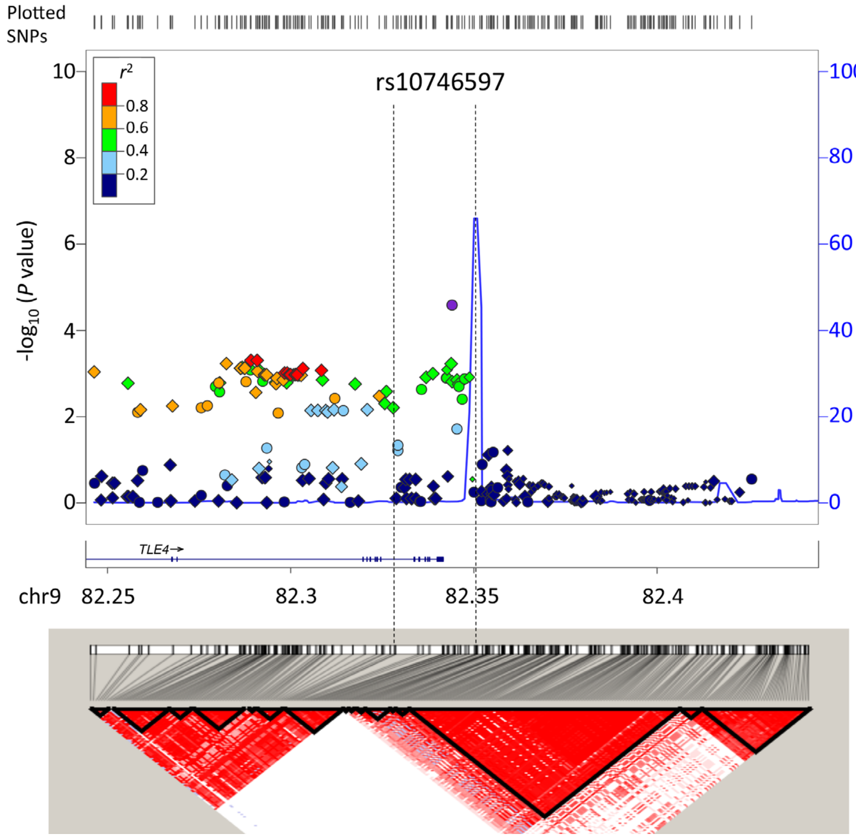

Supplementary Figure S3 (*Continued*): C. rs1532980, **D.** rs10746597

(*Continued*)

D

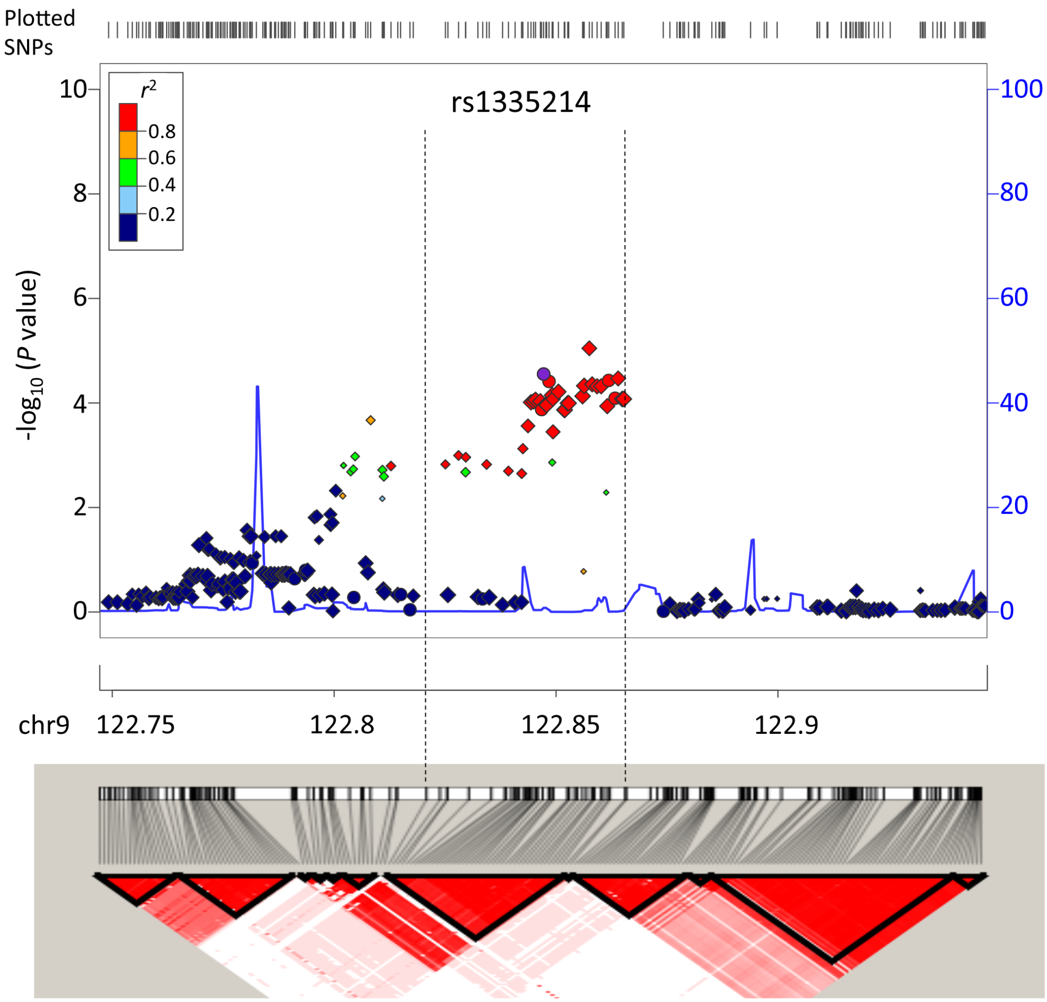

Supplementary Figure S3 (*Continued*): E. rs1335214. SNPs genotyped in this study or imputed on the basis of the 1000 Genomes Project data are indicated by circles or diamonds, respectively (top). Each point size is proportional to sample size. Symbol colors represent the LD of SNPs with the index SNP rs12567052, rs10235505, rs1532980, rs10746597 and rs1335214 (purple circles) at each locus respectively. The legend for LD measure (r^2) is on the top left corner of each regional association plot. The left Y-axis illustrates the $-\log_{10}$ association *P* values of SNPs and the Y-axis on the right shows the recombination rate estimated from the 1000 Genomes Project (Phase 1 integrated data version 3, March 2012) using Chinese and Japanese population data. The genome build 37/hg19 was used to annotate the SNP positions, recombination rates and genes. Plots were drawn using LocusZoom web-based tool (University of Michigan). The LD maps (bottom) are based on r^2 values in CHB and JPT samples from the 1000 Genomes Project and are constructed using Haploview software.

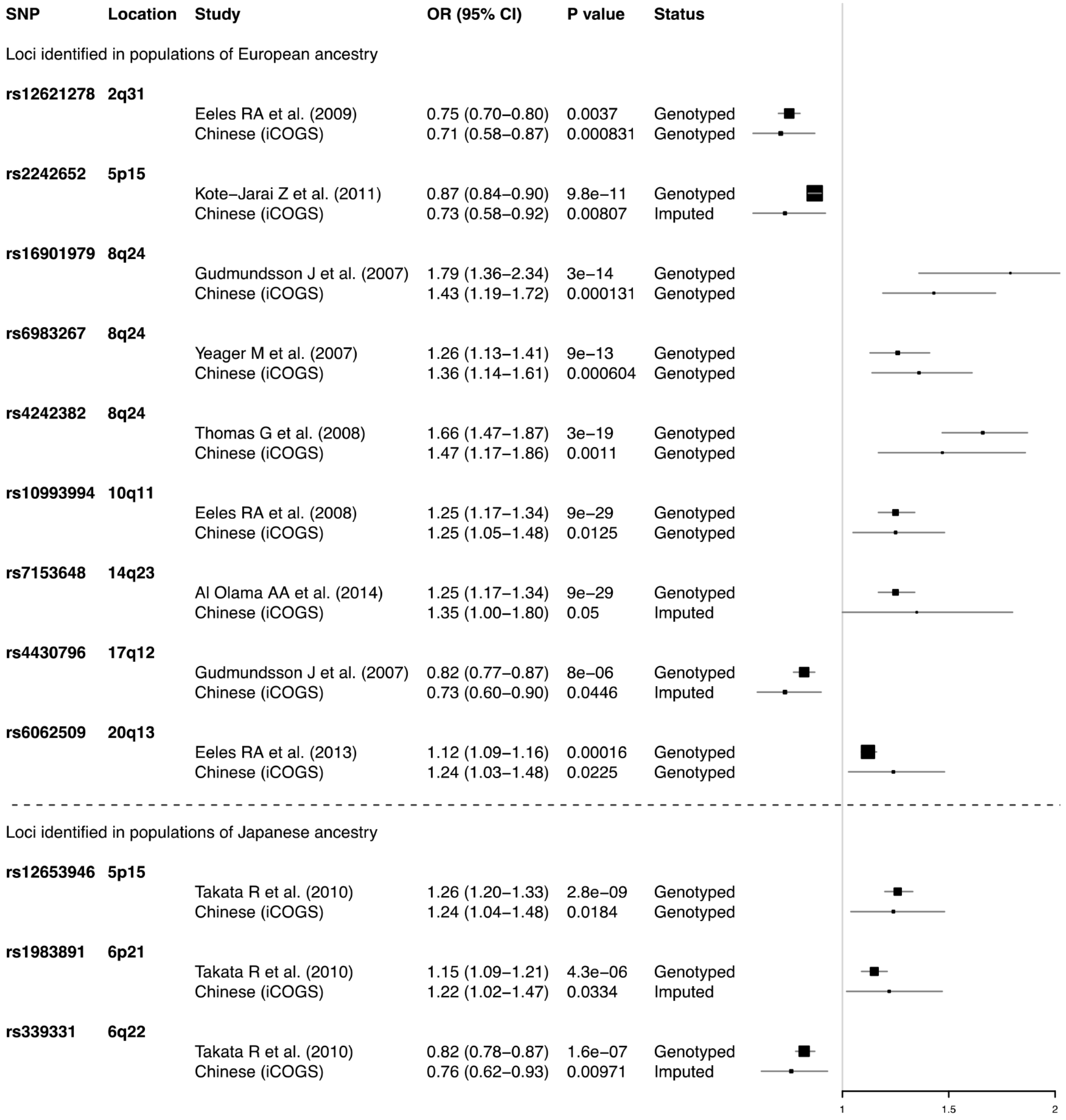

Supplementary Figure S4: Forest plot showing the point estimates and 95% confidence intervals of per-allele odds ratios for previously reported prostate cancer associated loci in Chinese sample iCOGS data (only for SNPs with *P*<0.05) and obtained at GWAS stage in published studies. The size of each square is inversely proportional to the variance of the estimate in individual study.

SNP	Location	Study	OR (95% CI)	P value	Status	Phet	I2
Loci identified in populations of European ancestry							
**rs636291**	**1p35**					**0.16**	**50.3%**
		Chinese (iCOGS)	1.11 (0.91–1.35)	0.32	Imputed		
		PRACTICAL (iCOGS)	0.96 (0.93–0.98)	0.00212	Imputed		
**rs17599629**	**1q21**					**0.99**	**0%**
		Chinese (iCOGS)	1.09 (0.80–1.50)	0.57	Imputed		
		PRACTICAL (iCOGS)	1.09 (1.06–1.13)	2.63e–06	Imputed		
**rs1218582**	**1q21**					**0.28**	**14.3%**
		Chinese (iCOGS)	1.12 (0.83–1.52)	0.47	Genotyped		
		PRACTICAL (iCOGS)	0.95 (0.92–0.98)	0.00023	Genotyped		
**rs4245739**	**1q32**					**0.62**	**0%**
		Chinese (iCOGS)	0.80 (0.48–1.35)	0.42	Genotyped		
		PRACTICAL (iCOGS)	0.92 (0.89–0.95)	7.4e–08	Genotyped		
**rs1775148**	**1q32**					**0.98**	**0%**
		Chinese (iCOGS)	0.92 (0.76–1.11)	0.37	Imputed		
		PRACTICAL (iCOGS)	1.05 (1.03–1.08)	0.000277	Imputed		
**rs11902236**	**2p25**					**0.98**	**0%**
		Chinese (iCOGS)	1.07 (0.82–1.38)	0.66	Genotyped		
		PRACTICAL (iCOGS)	1.07 (1.04–1.10)	2.5e–05	Genotyped		
**rs9287719**	**2p25**					**0.81**	**0%**
		Chinese (iCOGS)	1.04 (0.85–1.26)	0.7	Imputed		
		PRACTICAL (iCOGS)	1.06 (1.04–1.09)	8.31e–06	Imputed		
**rs1465618**	**2p21**					**0.46**	**0%**
		Chinese (iCOGS)	1.00 (0.82–1.21)	0.99	Genotyped		
		PRACTICAL (iCOGS)	0.93 (0.90–0.96)	4.8e–06	Genotyped		
**rs721048**	**2p15**					**0.89**	**0%**
		Chinese (iCOGS)	1.15 (0.74–1.79)	0.55	Genotyped		
		PRACTICAL (iCOGS)	1.12 (1.08–1.15)	3.7e–10	Genotyped		
**rs10187424**	**2p11**					**0.33**	**0%**
		Chinese (iCOGS)	1.00 (0.84–1.19)	0.97	Imputed		
		PRACTICAL (iCOGS)	0.91 (0.89–0.94)	5.2e–11	Imputed		
**rs12621278**	**2q31**					**0.58**	**0%**
		Chinese (iCOGS)	0.71 (0.58–0.87)	0.00083	Genotyped		
		PRACTICAL (iCOGS)	0.75 (0.69–0.81)	2.3e–19	Genotyped		
**rs7584330**	**2q37**					**0.08**	**67.9%**
		Chinese (iCOGS)	0.89 (0.73–1.08)	0.26	Genotyped		
		PRACTICAL (iCOGS)	1.07 (1.03–1.10)	0.00011	Genotyped		
**rs2292884**	**2q37**					**0.01**	**83.3%**
		Chinese (iCOGS)	0.84 (0.69–1.01)	0.07	Genotyped		
		PRACTICAL (iCOGS)	1.07 (1.03–1.10)	7.6e–05	Genotyped		
**rs3771570**	**2q37**					**0.39**	**0%**
		Chinese (iCOGS)	1.23 (0.95–1.60)	0.12	Genotyped		
		PRACTICAL (iCOGS)	1.10 (1.06–1.14)	1.1e–06	Genotyped		
**rs2660753**	**3p12**					**0.64**	**0%**
		Chinese (iCOGS)	1.18 (0.98–1.42)	0.08	Genotyped		
		PRACTICAL (iCOGS)	1.13 (1.09–1.17)	7.6e–08	Genotyped		
**rs7611694**	**3q13**					**0.83**	**0%**
		Chinese (iCOGS)	1.12 (0.93–1.35)	0.26	Genotyped		
		PRACTICAL (iCOGS)	1.09 (1.07–1.12)	1.4e–10	Genotyped		
**rs10934853**	**3q21**					**0.76**	**0%**
		Chinese (iCOGS)	1.15 (0.97–1.36)	0.12	Genotyped		
		PRACTICAL (iCOGS)	1.12 (1.09–1.15)	7e–13	Genotyped		
**rs6763931**	**3q23**					**0.34**	**0%**
		Chinese (iCOGS)	1.13 (0.94–1.36)	0.19	Genotyped		
		PRACTICAL (iCOGS)	1.03 (1.01–1.06)	0.014	Genotyped		
**rs10936632**	**3q26**					**0.71**	**0%**
		Chinese (iCOGS)	1.07 (0.89–1.30)	0.47	Imputed		
		PRACTICAL (iCOGS)	1.11 (1.09–1.14)	7.2e–15	Imputed		
**rs1894292**	**4q13**					**0.18**	**43.3%**
		Chinese (iCOGS)	1.03 (0.87–1.23)	0.76	Genotyped		
		PRACTICAL (iCOGS)	0.91 (0.89–0.94)	1.1e–10	Genotyped		
**rs12500426**	**4q22**					**0.03**	**79.2%**
		Chinese (iCOGS)	1.12 (0.94–1.34)	0.2	Genotyped		
		PRACTICAL (iCOGS)	0.92 (0.89–0.95)	9.6e–10	Genotyped		
**rs17021918**	**4q22**					**0.67**	**0%**
		Chinese (iCOGS)	0.94 (0.78–1.12)	0.49	Genotyped		
		PRACTICAL (iCOGS)	0.90 (0.87–0.93)	4.8e–13	Genotyped		
**rs7679673**	**4q24**					**0.17**	**46.1%**
		Chinese (iCOGS)	0.99 (0.80–1.24)	0.98	Genotyped		
		PRACTICAL (iCOGS)	1.16 (1.13–1.18)	1.9e–24	Genotyped		

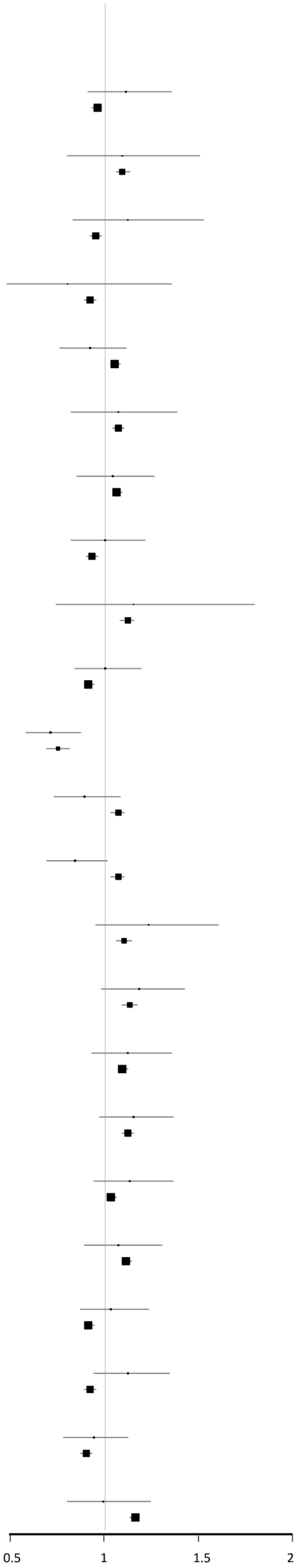

(Continued)

SNP	Locus	Study	OR (95% CI)	P	Type	P het	I²
rs2242652	5p15					0.23	30.1%
		Chinese (iCOGS)	0.73 (0.58–0.92)	0.01	Imputed		
		PRACTICAL (iCOGS)	0.84 (0.83–0.86)	0	Imputed		
rs2121875	5p12					0.57	0%
		Chinese (iCOGS)	0.98 (0.83–1.17)	0.86	Imputed		
		PRACTICAL (iCOGS)	0.94 (0.91–0.96)	5e−06	Imputed		
rs6869841	5q35					0.97	0%
		Chinese (iCOGS)	1.08 (0.88–1.33)	0.46	Genotyped		
		PRACTICAL (iCOGS)	1.09 (1.06–1.12)	6.2e−07	Genotyped		
rs4713266	6p24					0.31	4.8%
		Chinese (iCOGS)	1.20 (0.96–1.49)	0.11	Imputed		
		PRACTICAL (iCOGS)	1.07 (1.04–1.09)	2.01e−06	Imputed		
rs115457135	6p22					0.96	0%
		Chinese (iCOGS)	1.08 (0.89–1.32)	0.41	Imputed		
		PRACTICAL (iCOGS)	1.09 (1.06–1.12)	2.5e−07	Imputed		
rs130067	6p21					0.94	0%
		Chinese (iCOGS)	1.08 (0.89–1.30)	0.45	Genotyped		
		PRACTICAL (iCOGS)	1.07 (1.04–1.10)	7.1e−05	Genotyped		
rs3096702	6p21					0.76	0%
		Chinese (iCOGS)	1.11 (0.88–1.39)	0.4	Genotyped		
		PRACTICAL (iCOGS)	1.07 (1.04–1.10)	7.1e−05	Genotyped		
rs115306967	6p21					0.68	0%
		Chinese (iCOGS)	0.89 (0.71–1.10)	0.27	Imputed		
		PRACTICAL (iCOGS)	0.93 (0.90–0.96)	2.29e−07	Imputed		
rs9443189	6q14					0.72	0%
		Chinese (iCOGS)	0.91 (0.76–1.08)	0.27	Imputed		
		PRACTICAL (iCOGS)	0.94 (0.90–0.97)	0.000808	Imputed		
rs2273669	6q21					0.58	0%
		Chinese (iCOGS)	1.19 (0.85–1.65)	0.32	Genotyped		
		PRACTICAL (iCOGS)	1.08 (1.04–1.12)	6.6e−05	Genotyped		
rs1933488	6q25					0.37	0%
		Chinese (iCOGS)	1.24 (0.99–1.56)	0.06	Genotyped		
		PRACTICAL (iCOGS)	1.12 (1.09–1.14)	3.3e−15	Genotyped		
rs651164	6q25					0.61	0%
		Chinese (iCOGS)	1.09 (0.92–1.30)	0.3	Genotyped		
		PRACTICAL (iCOGS)	1.14 (1.11–1.17)	3.63e−19	Genotyped		
rs9364554	6q25					0.43	0%
		Chinese (iCOGS)	1.03 (0.86–1.22)	0.82	Genotyped		
		PRACTICAL (iCOGS)	1.10 (1.07–1.13)	6.4e−11	Genotyped		
rs12155172	7p15					0.4	0%
		Chinese (iCOGS)	1.02 (0.84–1.22)	0.9	Genotyped		
		PRACTICAL (iCOGS)	1.10 (1.07–1.13)	2.8e−09	Genotyped		
rs10486567	7p15					0.15	50.6%
		Chinese (iCOGS)	0.96 (0.75–1.22)	0.75	Genotyped		
		PRACTICAL (iCOGS)	1.15 (1.11–1.18)	2.5e−16	Genotyped		
rs56232506	7p12					0.23	32.0%
		Chinese (iCOGS)	1.20 (1.00–1.44)	0.06	Imputed		
		PRACTICAL (iCOGS)	1.07 (1.04–1.09)	2.06e−06	Imputed		
rs6465657	7q21					0.43	0%
		Chinese (iCOGS)	0.99 (0.78–1.26)	0.97	Genotyped		
		PRACTICAL (iCOGS)	0.90 (0.87–0.92)	2.7e−15	Genotyped		
rs2928679	8p21					0.17	46.9%
		Chinese (iCOGS)	1.26 (0.98–1.62)	0.08	Genotyped		
		PRACTICAL (iCOGS)	1.05 (1.03–1.08)	2e−04	Genotyped		
rs1512268	8p21					0.82	0%
		Chinese (iCOGS)	1.11 (0.93–1.34)	0.27	Genotyped		
		PRACTICAL (iCOGS)	1.14 (1.10–1.18)	3.3e−20	Genotyped		
rs11135910	8p21					0.5	0%
		Chinese (iCOGS)	1.30 (0.81–2.09)	0.29	Genotyped		
		PRACTICAL (iCOGS)	1.11 (1.07–1.14)	1.4e−07	Genotyped		
rs12543663	8q24					0.67	0%
		Chinese (iCOGS)	1.05 (0.72–1.54)	0.79	Genotyped		
		PRACTICAL (iCOGS)	1.14 (1.11–1.17)	5.09e−19	Genotyped		
rs10086908	8q24					0.9	0%
		Chinese (iCOGS)	0.86 (0.68–1.08)	0.2	Genotyped		
		PRACTICAL (iCOGS)	0.87 (0.84–0.90)	7e−20	Genotyped		
rs16901979	8q24					0.14	54.7%
		Chinese (iCOGS)	1.43 (1.19–1.72)	0.00013	Genotyped		
		PRACTICAL (iCOGS)	1.66 (1.59–1.74)	1.1e−45	Genotyped		
rs16902094	8q24					0.03	77.6%
		Chinese (iCOGS)	0.97 (0.80–1.18)	0.8	Genotyped		
		PRACTICAL (iCOGS)	1.21 (1.17–1.24)	3e−23	Genotyped		

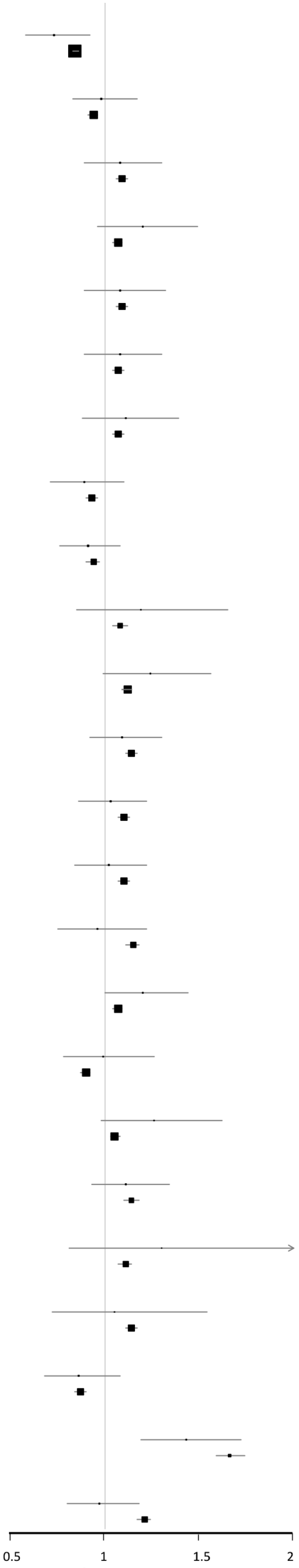

(*Continued*)

**rs445114**	**8q24**					**0.85**	**0%**
		Chinese (iCOGS)	0.89 (0.75–1.06)	0.18	Genotyped		
		PRACTICAL (iCOGS)	0.87 (0.84–0.90)	5.7e–21	Genotyped		
**rs620861**	**8q24**					**0.41**	**0%**
		Chinese (iCOGS)	0.93 (0.79–1.11)	0.46	Genotyped		
		PRACTICAL (iCOGS)	0.87 (0.84–0.90)	4.1e–22	Genotyped		
**rs6983267**	**8q24**					**0.39**	**0%**
		Chinese (iCOGS)	1.36 (1.14–1.61)	0.000604	Genotyped		
		PRACTICAL (iCOGS)	1.25 (1.22–1.28)	2e–57	Genotyped		
**rs4242382**	**8q24**					**0.25**	**26%**
		Chinese (iCOGS)	1.65 (1.31–2.07)	2.01e–05	Genotyped		
		PRACTICAL (iCOGS)	1.44 (1.40–1.48)	6.61e–68	Genotyped		
**rs17694493**	**9p21**					**0.29**	**10.8%**
		Chinese (iCOGS)	0.78 (0.43–1.42)	0.42	Genotyped		
		PRACTICAL (iCOGS)	1.08 (1.04–1.12)	9.4e–05	Genotyped		
**rs1571801**	**9q33**					**0.64**	**0%**
		Chinese (iCOGS)	1.15 (0.76–1.75)	0.52	Genotyped		
		PRACTICAL (iCOGS)	1.04 (1.01–1.08)	0.0046	Genotyped		
**rs10993994**	**10q11**					**0.89**	**0%**
		Chinese (iCOGS)	1.25 (1.05–1.48)	0.01	Genotyped		
		PRACTICAL (iCOGS)	1.23 (1.20–1.26)	7.2e–49	Genotyped		
**rs3850699**	**10q24**					**0.5**	**0%**
		Chinese (iCOGS)	1.00 (0.78–1.29)	0.98	Genotyped		
		PRACTICAL (iCOGS)	0.92 (0.89–0.95)	3e–08	Genotyped		
**rs4962416**	**10q26**					**0.99**	**0%**
		Chinese (iCOGS)	1.05 (0.41–2.68)	0.92	Imputed		
		PRACTICAL (iCOGS)	1.04 (1.01–1.07)	0.00575	Imputed		
**rs7127900**	**11p15**					**0.33**	**0%**
		Chinese (iCOGS)	1.08 (0.82–1.41)	0.61	Genotyped		
		PRACTICAL (iCOGS)	1.23 (1.20–1.27)	5.6e–35	Genotyped		
**rs12418451**	**11q13**					**0.22**	**34.6%**
		Chinese (iCOGS)	0.92 (0.65–1.29)	0.64	Genotyped		
		PRACTICAL (iCOGS)	1.14 (1.11–1.17)	6.4e–18	Genotyped		
**rs11228565**	**11q13**					**0.15**	**51.2%**
		Chinese (iCOGS)	0.93 (0.63–1.36)	0.71	Genotyped		
		PRACTICAL (iCOGS)	1.23 (1.20–1.26)	1.04e–34	Genotyped		
**rs7931342**	**11q13**					**0.02**	**82.3%**
		Chinese (iCOGS)	0.94 (0.77–1.14)	0.55	Genotyped		
		PRACTICAL (iCOGS)	1.19 (1.17–1.22)	1.5e–37	Genotyped		
**rs10896449**	**11q13**					**0.29**	**11.6%**
		Chinese (iCOGS)	1.01 (0.74–1.38)	0.97	Genotyped		
		PRACTICAL (iCOGS)	1.20 (1.17–1.23)	2.9e–39	Genotyped		
**rs11568818**	**11q22**					**0.47**	**0%**
		Chinese (iCOGS)	1.01 (0.74–1.37)	0.95	Genotyped		
		PRACTICAL (iCOGS)	0.90 (0.87–0.93)	1.8e–13	Genotyped		
**rs11214775**	**11q23**					**0.45**	**0%**
		Chinese (iCOGS)	1.05 (0.75–1.47)	0.79	Imputed		
		PRACTICAL (iCOGS)	0.92 (0.87–0.95)	3e–07	Imputed		
**rs10875943**	**12q13**					**0.35**	**0%**
		Chinese (iCOGS)	0.80 (0.63–1.04)	0.09	Genotyped		
		PRACTICAL (iCOGS)	0.91 (0.88–0.94)	1.5e–10	Genotyped		
**rs902774**	**12q13**					**0.16**	**48.9%**
		Chinese (iCOGS)	2.45 (0.82–7.37)	0.11	Imputed		
		PRACTICAL (iCOGS)	1.12 (1.08–1.15)	1.09e–09	Imputed		
**rs1270884**	**12q24**					**0.17**	**47.4%**
		Chinese (iCOGS)	0.94 (0.78–1.14)	0.54	Genotyped		
		PRACTICAL (iCOGS)	1.08 (1.05–1.10)	1.6e–07	Genotyped		
**rs7153648**	**14q23**					**0.24**	**26.7%**
		Chinese (iCOGS)	1.35 (1.00–1.80)	0.05	Imputed		
		PRACTICAL (iCOGS)	1.13 (1.06–1.19)	0.000136	Imputed		
**rs7141529**	**14q24**					**0.44**	**0%**
		Chinese (iCOGS)	0.85 (0.68–1.06)	0.15	Genotyped		
		PRACTICAL (iCOGS)	0.92 (0.90–0.95)	1.4e–08	Genotyped		
**rs12051443**	**16q22**					**0.59**	**0%**
		Chinese (iCOGS)	0.90 (0.73–1.10)	0.3	Imputed		
		PRACTICAL (iCOGS)	0.95 (0.92–0.98)	0.000269	Imputed		
**rs684232**	**17p13**					**0.68**	**0%**
		Chinese (iCOGS)	0.88 (0.74–1.04)	0.14	Genotyped		
		PRACTICAL (iCOGS)	0.91 (0.88–0.94)	1.9e–11	Genotyped		
**rs11649743**	**17q12**					**0.55**	**0%**
		Chinese (iCOGS)	0.94 (0.79–1.12)	0.5	Genotyped		
		PRACTICAL (iCOGS)	0.89 (0.85–0.92)	2.6e–11	Genotyped		

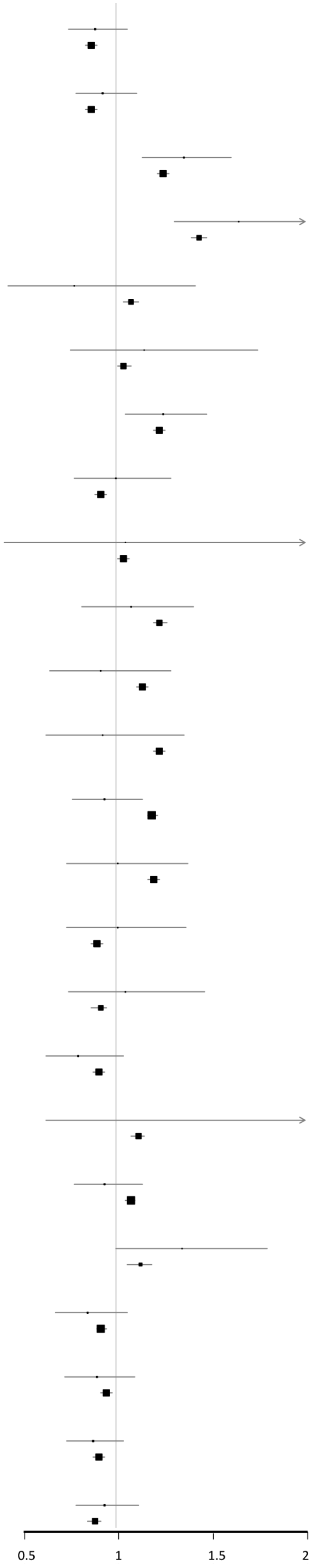

(*Continued*)

**rs4430796**	**17q12**					**0.36**	**0%**
		Chinese (iCOGS)	0.73 (0.60–0.90)	0.0033	Imputed		
		PRACTICAL (iCOGS)	0.81 (0.79–0.82)	0	Imputed		
**rs11650494**	**17q21**					**0.41**	**0%**
		Chinese (iCOGS)	1.67 (0.68–4.06)	0.26	Imputed		
		PRACTICAL (iCOGS)	1.14 (1.09–1.19)	1.56e–07	Imputed		
**rs1859962**	**17q24**					**0.17**	**45.8%**
		Chinese (iCOGS)	1.05 (0.88–1.25)	0.59	Genotyped		
		PRACTICAL (iCOGS)	1.19 (1.16–1.22)	5.5e–36	Genotyped		
**rs7241993**	**18q23**					**0.38**	**0%**
		Chinese (iCOGS)	1.00 (0.85–1.19)	0.97	Genotyped		
		PRACTICAL (iCOGS)	0.93 (0.90–0.96)	6.6e–07	Genotyped		
**rs8102476**	**19q13**					**0.75**	**0%**
		Chinese (iCOGS)	1.05 (0.88–1.25)	0.65	Genotyped		
		PRACTICAL (iCOGS)	1.08 (1.05–1.10)	1.5e–07	Genotyped		
**rs11672691**	**19q13**					**0.18**	**44.0%**
		Chinese (iCOGS)	1.03 (0.86–1.22)	0.77	Genotyped		
		PRACTICAL (iCOGS)	0.90 (0.87–0.93)	2.4e–11	Genotyped		
**rs887391**	**19q13**					**0.1**	**64.0%**
		Chinese (iCOGS)	1.05 (0.88–1.25)	0.64	Genotyped		
		PRACTICAL (iCOGS)	0.91 (0.94–0.88)	6.2e–09	Genotyped		
**rs2735839**	**19q13**					**0.03**	**79.5%**
		Chinese (iCOGS)	1.04 (0.87–1.24)	0.74	Genotyped		
		PRACTICAL (iCOGS)	0.84 (0.80–0.88)	1.3e–16	Genotyped		
**rs12480328**	**20q13**					**0.65**	**0%**
		Chinese (iCOGS)	0.83 (0.56–1.16)	0.27	Imputed		
		PRACTICAL (iCOGS)	0.89 (0.84–0.95)	3.77e–05	Imputed		
**rs2427345**	**20q13**					**0.02**	**81.2%**
		Chinese (iCOGS)	1.22 (0.97–1.53)	0.09	Genotyped		
		PRACTICAL (iCOGS)	0.93 (0.91–0.96)	1.5e–06	Genotyped		
**rs6062509**	**20q13**					**0.27**	**17.1%**
		Chinese (iCOGS)	1.24 (1.03–1.48)	0.02	Genotyped		
		PRACTICAL (iCOGS)	1.12 (1.09–1.14)	3.9e–13	Genotyped		
**rs1041449**	**21q22**					**0.58**	**0%**
		Chinese (iCOGS)	1.14 (0.92–1.42)	0.24	Genotyped		
		PRACTICAL (iCOGS)	1.07 (1.04–1.10)	1.13e–06	Genotyped		
**rs2238776**	**22q11**					**0.77**	**0%**
		Chinese (iCOGS)	0.91 (0.75–1.10)	0.33	Imputed		
		PRACTICAL (iCOGS)	0.93 (0.90–0.97)	0.000299	Imputed		
**rs9623117**	**22q13**					**0.42**	**0%**
		Chinese (iCOGS)	0.89 (0.55–1.43)	0.64	Genotyped		
		PRACTICAL (iCOGS)	1.08 (1.04–1.11)	8.2e–06	Genotyped		
**rs5759167**	**22q13**					**0.01**	**84.1%**
		Chinese (iCOGS)	1.11 (0.91–1.35)	0.29	Imputed		
		PRACTICAL (iCOGS)	0.87 (0.85–0.88)	0	Imputed		
**rs2405942**	**Xp22**					**0.6**	**0%**
		Chinese (iCOGS)	0.84 (0.55–1.26)	0.41	Genotyped		
		PRACTICAL (iCOGS)	0.93 (0.91–0.96)	2.1e–08	Genotyped		
**rs5945572**	**Xp11**					**0.97**	**0%**
		Chinese (iCOGS)	1.10 (0.70–1.72)	0.72	Genotyped		
		PRACTICAL (iCOGS)	1.11 (1.09–1.13)	5.7e–24	Genotyped		
**rs5945619**	**Xp11**					**0.9**	**0%**
		Chinese (iCOGS)	1.08 (0.69–1.68)	0.78	Genotyped		
		PRACTICAL (iCOGS)	1.11 (1.09–1.13)	2.6e–24	Genotyped		

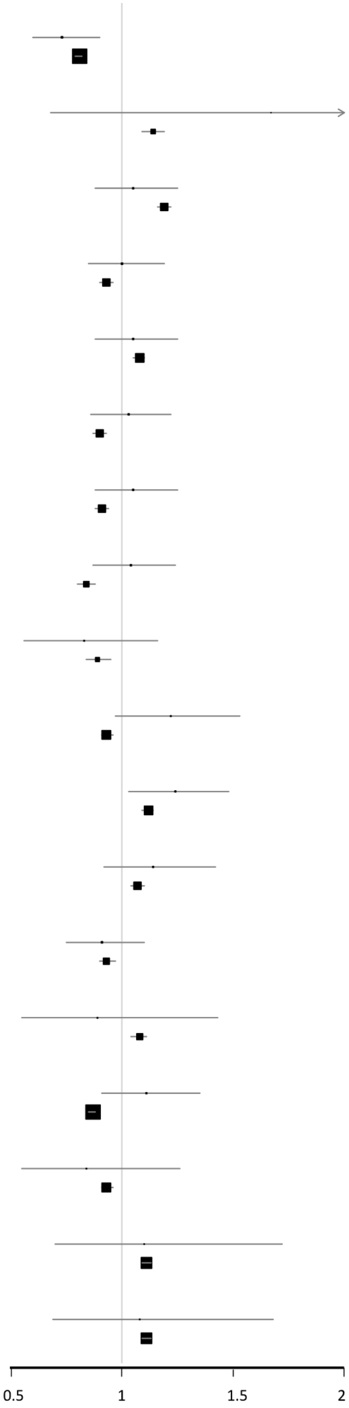

(Continued)

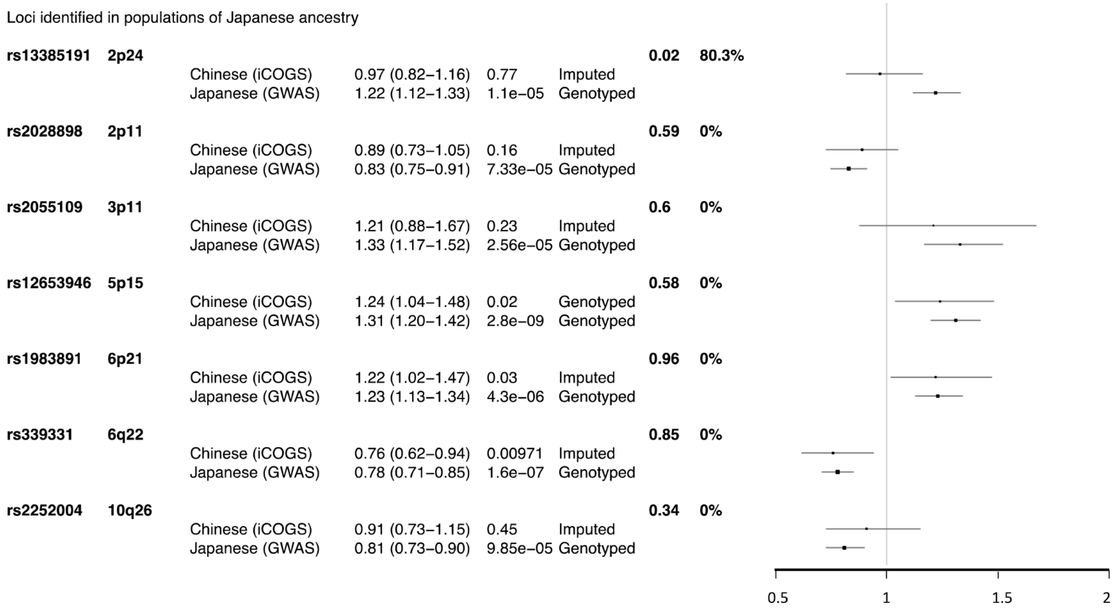

Supplementary Figure S5: Forest plot showing the point estimates and 95% confidence intervals of per-allele odds ratios for previously reported prostate cancer associated loci. Presented OR and *P* values for prostate cancer associated loci identified in European populations were computed for iCOGS data sets from Chinese and European (PRACTICAL) descendants. The OR ratios and *P* values for prostate cancer associated loci identified in Japanese populations were computed using iCOGS data from Chinese population and published Japanese GWAS data. P_{het} and I^2 values indicate Cochran's Q statistic and index of heterogeneity, respectively, estimated for individual loci between corresponding data sets.

Supplementary Table S1: Study subjects characterization

	iCOGS		Replication	
**Parameters**	**Cases (n=495)**	**Controls (n=640)**	**Cases (n=1940)**	**Controls (n=2820)**
Age mean (SD)	68.76 (9.11)	70.52 (7.88)	71.2 (8.21)	64.46 (11.79)
PSA levels (ng/ml)				
≤9.99	61 (22.6%)		352 (30%)	
10-19.99	71 (26.3%)		318 (20.7%)	
20-49.99	47 (17.4%)		301 (19.6%)	
≥50	91 (33.7%)		563 (36.7%)	
Gleason score				
<7	74 (38.9%)		365 (27.7%)	
=7	70 (36.8%)		514 (39.1%)	
>7	46 (24.2%)		437 (33.2%)	
T-stage				
T1	27 (22.7%)		125 (16.1%)	
T2	41 (34.5%)		389 (50.1%)	
T3	32 (26.9%)		223 (28.7%)	
T4	19 (16%)		39 (5%)	
N-stage				
N0	94 (81.7%)		773 (92.5%)	
N1	21 (18.3%)		63 (7.5%)	
M-stage				
M0	95 (66.4%)		732 (73.9%)	
M1	48 (33.6%)		258 (26.1%)	

Abbreviations: SD, standard deviation; PSA, prostate-specific antigen.

Supplementary Table S2: Multivariate stepwise logistic regression analysis results for three 8q24 loci with $P<1\times10^{-4}$ and previously reported prostate cancer predisposition 8q24 loci

SNP	Location	Allele[a]	Position	OR (95% CI)[b]	P[c]
rs12543663[d]	8q24	A/C	127924659		
rs10086908[d]	8q24 (5)	T/C	128011937		
rs7463708	8q24 (2?)	T/G	128104055	0.65 (0.52-0.81)	1.2×10^{-4}
rs16901979	8q24 (2)	C/A	128124916	1.21 (0.99-1.48)	0.06
rs7013255[d]	8q24 (2)	T/G	128130487		
rs16902094[d]	8q24	A/G	128320346		
rs445114	8q24	T/C	128323181	0.83 (0.69-1.00)	0.05
rs620861[d]	8q24 (4)	C/T	128335673		
rs6983267	8q24 (3)	T/G	128413305	1.38 (1.15-1.66)	4.59×10^{-4}
rs4242382[d]	8q24 (1)	G/A	128517573		
rs13255059	8q24 (1)	G/A	128530616	1.66 (1.31-2.12)	2.94×10^{-5}

[a]Major/minor allele based on the frequencies in iCOGS data from Chinese population.
[b]Odds ratio values with 95% confidence intervals, adjusted for effects of the remaining SNPs in the multivariate model, calculated for minor alleles.
[c]*P* value adjusted for effects of the remaining SNPs in the multivariate model.
[d]SNPs excluded by the stepwise procedure (rs12543663, rs10086908, rs7013255, rs16902094, rs620861 and rs4242382).

Supplementary Table S3: Chinese iCOGS data association results for previously reported prostate cancer susceptibility loci in populations of European and Japanese ancestry

See Supplementary File: 1

Supplementary Table S4: Meta-analysis of Chinese iCOGS and Chinese GWAS data using SNPs associated with prostate cancer with P<0.001 in Chinese iCOGS array data

See Supplementary File: 1

Supplementary Table S5: Odds ratios for prostate cancer by percentile of the polygenic risk score

	Percentile	OR (95% CI)[a,b]	OR (95% CI)[a,c]
**PRS group**	< 10%	1 (baseline)	0.33 (0.16-0.70)
	10-25%	1.78 (0.80-3.97)	0.59 (0.40-0.87)
	25-75%	3.02 (1.43-6.37)	1 (baseline)
	75-90%	4.26 (1.97-9.19)	1.41 (1.03-1.94)
	>90%	5.91 (2.68-13.04)	1.96 (1.36-2.82)

Abbreviation: PRS, polygenic risk score.
[a]Odds ratio (OR) values obtained by fitting PRS group.
[b]ORs with 95% confidence intervals (CI) compared to men in the 10th percentile as baseline.
[c]ORs with 95% confidence intervals (CI) compared to men in the 25th-75th percentile as baseline.

Supplementary Table S6: Results summary at iCOGS array and replication stages for unreported prostate cancer susceptibility loci with $P<1\times10^{-4}$ in iCOGS array data

SNP	Location[a]	Alleles[b]	Gene	Study	MAF Case	MAF Control	OR (95% CI)[c]	P_{GC}[d]
rs12567052	1q32.1 (200494416)	G/A	*KIF14*	iCOGS	0.241	0.321	0.67 (0.55-0.81)	5.89×10^{-5}
				Replication	0.250	0.288	0.83 (0.75-0.91)	1.85×10^{-4}
				Combined			0.79 (0.72-0.86)	2.41×10^{-7}
rs10235505	7p21.3 (7441152)	G/A	*COL28A1*	iCOGS	0.347	0.266	1.45 (1.20-1.74)	9.09×10^{-5}
				Replication	0.313	0.279	1.17 (1.07-1.29)	9.86×10^{-4}
				Combined			1.22 (1.12-1.33)	2.44×10^{-6}
rs1532980	8p23.1 (9848617)	T/C		iCOGS	0.428	0.486	0.70 (0.59-0.84)	8.65×10^{-5}
				Replication	0.469	0.451	1.07 (0.98-1.18)	1.42×10^{-1}
				Combined			0.98 (0.90-1.06)	5.65×10^{-1}
rs10746597	9q21.31 (82344077)	A/G	*TLE4*	iCOGS	0.354	0.271	1.52 (1.25-1.85)	2.58×10^{-5}
				Replication	0.285	0.314	0.87 (0.78-0.97)	1.04×10^{-2}
				Combined			0.99 (0.90-1.09)	8.13×10^{-1}
rs1335214	9q33.2 (122847182)	G/C		iCOGS	0.467	0.376	1.46 (1.22-1.74)	2.82×10^{-5}
				Replication	0.437	0.415	1.09 (0.99-1.20)	7.88×10^{-2}
				Combined			1.16 (1.07-1.26)	4.26×10^{-4}

Abbreviation: MAF, minor allele frequency.
[a]Chromosomal and physical (in bracket) locations based on NCBI Human Genome Build 37.
[b]Major/minor allele.
[c]Odds ratio values with 95% confidence intervals for the minor allele in association with prostate cancer risk.
[d]P_{GC} values at iCOGS array stage indicate λ-corrected P values.

Supplementary Table S7: SNPs used for polygenic risk score calculation

SNP	Location[a]	Alleles[b]	OR (95% CI)[c]	*P*[d]
rs1456315	128103937	A/G	1.64 (1.47-1.82)	7.93×10^{-18}
rs16901979	128124916	C/A	1.42 (1.28-1.57)	4.69×10^{-11}
rs6983267	128413305	T/G	1.28 (1.16-1.41)	4.16×10^{-7}
rs4242382	128517573	G/A	1.55 (1.37-1.76)	1.15×10^{-11}

[a]Physical locations based on NCBI Human Genome Build 37.
[b]Major/minor allele.
[c]Odds ratio values with 95% confidence intervals for the risk allele estimated in meta-analysis.
[d]Combined *P* value.
