# Supplementary material for: A genetic study and meta-analysis of the genetic predisposition of prostate cancer in a Chinese population
Source: Oncotarget. 2016 Feb 8;7(16):21393–403. doi: 10.18632/oncotarget.7250 (PMC5008293; doi:10.18632/oncotarget.7250)
Supplement: Supplementary file 2 [file oncotarget-07-21393-s002.docx]

**Supplementary Table S3. Chinese iCOGS data association results for previously reported prostate cancer susceptibility loci in populations of European and Japanese ancestry.**

|  |  |  |  |  |  | **Chinese iCOGS** | |  | **PRACTICAL iCOGS** | |  | **Heterogeneity** | |
| --- | --- | --- | --- | --- | --- | --- | --- | --- | --- | --- | --- | --- | --- |
| **SNP** | **Location** | **Allele^a^** | **Position** | **Nearby gene** | **Status** | **OR (95% CI)^b^** | ***P*** |  | **OR (95% CI)^b^** | ***P*** |  | ***P*_het_^c^** | ***I*^2d^** |
| Loci identified in populations of European ancestry | | | | |  |  | |  |  | |  |  | |
| rs636291 | 1p35 | A/G | 10556097 | *PEX14* | Imputed | 1.11 (0.91-1.35) | 0.32 |  | 0.96 (0.93-0.98) | 2.12×10^-3^ |  | 0.16 | 50.3% |
| rs17599629 | 1q21 | A/G | 150658287 | *GOLPH3L* | Imputed | 1.09 (0.80-1.50) | 0.57 |  | 1.09 (1.06-1.13) | 2.63×10^-7^ |  | 0.99 | 0% |
| rs1218582 | 1q21 | C/T | 154834183 | *KCNN3* | Genotyped | 1.12 (0.83-1.52) | 0.47 |  | 0.95 (0.92-0.98) | 2.25×10^-4^ |  | 0.28 | 14.3% |
| rs4245739 | 1q32 | A/C | 204518842 | *MDM4* | Genotyped | 0.80 (0.48-1.35) | 0.42 |  | 0.92 (0.89-0.95) | 7.43×10^-^**^8^** |  | 0.62 | 0% |
| rs1775148  **rs9287719** | 1q32 | T/C | 205757824 | *SLC41A1* | Imputed | 0.92 (0.76-1.11) | 0.37 |  | 1.05 (1.03-1.08) | 2.77×10^-4^ |  | 0.15 | 51.7% |
| rs11902236 | 2p25 | C/T | 10117868 | *GRHL1* | Genotyped | 1.07 (0.82-1.38) | 0.66 |  | 1.07 (1.04-1.10) | 2.55×10^-^**^5^** |  | 0.98 | 0% |
| rs9287719 | 2p25 | T/C | 10710730 | *NOL10* | Imputed | 1.04 (0.85-1.26) | 0.7 |  | 1.06 (1.04-1.09) | 8.31×10^-^**^6^** |  | 0.81 | 0% |
| rs1465618 | 2p21 | A/G | 43553949 | *THADA* | Genotyped | 1.00 (0.82-1.21) | 0.99 |  | 0.93 (0.90-0.96) | 4.84×10^-^**^6^** |  | 0.46 | 0% |
| rs721048 | 2p15 | G/A | 63131731 | *EHBP1* | Genotyped | 1.15 (0.74-1.79) | 0.55 |  | 1.12 (1.08-1.15) | 3.72×10^-^**^10^** |  | 0.89 | 0% |
| rs10187424 | 2p11 | T/C | 85794297 | *GGCX*/*VAMP8* | Imputed | 1.00 (0.84-1.19) | 0.97 |  | 0.91 (0.89-0.94) | 5.21×10^-^**^11^** |  | 0.33 | 0% |
| *rs12621278* | *2q31* | A/G | *173311553* | *ITGA6* | Genotyped | *0.71 (0.58-0.87)* | *8.31×10^-4^* |  | *0.75 (0.69-0.81)* | *2.25*×*10^-19^* |  | *0.58* | *0%* |
| rs7584330 | 2q37 | A/G | 238387228 | *MLPH* | Genotyped | 0.89 (0.73-1.08) | 0.26 |  | 1.07 (1.03-1.10) | 1.05×10^-4^ |  | 0.08 | 67.9% |
| **rs2292884** | **2q37** | **A/G** | **238443226** | ***MLPH*** | **Genotyped** | **0.84 (0.69-1.01)** | **0.07** |  | **1.07 (1.03-1.10)** | **7.59×10^-5^** |  | **0.01** | **83.3%** |
| rs3771570 | 2q37 | C/T | 242382864 | *FARP2* | Genotyped | 1.23 (0.95-1.60) | 0.12 |  | 1.10 (1.06-1.14) | 1.07×10^-^**^6^** |  | 0.39 | 0% |
| rs2660753 | 3p12 | C/T | 87110674 |  | Genotyped | 1.18 (0.98-1.42) | 0.08 |  | 1.13 (1.09-1.17) | 7.55×10^-^**^8^** |  | 0.64 | 0% |
| rs7611694 | 3q13 | C/A | 113275624 | *SIDT1* | Genotyped | 1.12 (0.93-1.35) | 0.26 |  | 1.09 (1.07-1.12) | 1.39×10^-^**^10^** |  | 0.83 | 0% |
| rs10934853 | 3q21 | C/A | 128038373 | *EEFSEC* | Genotyped | 1.15 (0.97-1.36) | 0.12 |  | 1.12 (1.09-1.15) | 7.03×10^-^**^13^** |  | 0.76 | 0% |
| rs6763931 | 3q23 | G/A | 141102833 | *ZBTB38* | Genotyped | 1.13 (0.94-1.36) | 0.19 |  | 1.03 (1.01-1.06) | 0.01 |  | 0.34 | 0% |
| rs10936632 | 3q26 | C/A | 170130102 | *CLDN11*/*SKIL* | Imputed | 1.07 (0.89-1.30) | 0.47 |  | 1.11 (1.09-1.14) | 7.22×10^-^**^15^** |  | 0.71 | 0% |
| rs10009409 | 4q13 |  | 73855253 | *COX19* | N/A**^e^** |  |  |  |  |  |  |  |  |
| rs1894292 | 4q13 | G/A | 74349158 | *AFM* | Genotyped | 1.03 (0.87-1.23) | 0.76 |  | 0.91 (0.89-0.94) | 1.07×10^-10^ |  | 0.18 | 43.3% |
| **rs12500426** | **4q22** | **A/C** | **95514609** | ***PDLIM5*** | **Genotyped** | **1.12 (0.94-1.34)** | **0.2** |  | **0.92 (0.89-0.95)** | **9.55×10^-10^** |  | **0.03** | **79.2%** |
| rs17021918 | 4q22 | C/T | 95562877 | *PDLIM5* | Genotyped | 0.94 (0.78-1.12) | 0.49 |  | 0.90 (0.87-0.93) | 4.82×10^-^**^13^** |  | 0.67 | 0% |
| rs7679673 | 4q24 | A/C | 106061534 | *TET2* | Genotyped | 0.99 (0.80-1.24) | 0.98 |  | 1.16 (1.13-1.18) | 1.90×10^-24^ |  | 0.17 | 46.1% |
| *rs2242652* | *5p15* | G/A | *1280028* | *TERT* | Imputed | *0.73 (0.58-0.92)* | *8.07×10^-3^* |  | *0.84 (0.83-0.86)* | *0* |  | *0.23* | *30.1%* |
| rs2121875 | 5p12 | C/A | 44365545 | *FGF10* | Imputed | 0.98 (0.83-1.17) | 0.86 |  | 0.94 (0.91-0.96) | 5.00×10^-^**^6^** |  | 0.57 | 0% |
| rs6869841 | 5q35 | C/T | 172939426 |  | Genotyped | 1.08 (0.88-1.33) | 0.46 |  | 1.09 (1.06-1.12) | 6.17×10^-^**^7^** |  | 0.97 | 0% |
| rs4713266 | 6p24 | T/C | 11219030 | *NEDD9* | Imputed | 1.20 (0.96-1.49) | 0.11 |  | 1.07 (1.04-1.09) | 2.01×10^-^**^6^** |  | 0.31 | 4.8% |
| rs115457135 | 6p22 | G/A | 30073776 | *TRIM31* | Imputed | 1.08 (0.89-1.32) | 0.41 |  | 1.09 (1.06-1.12) | 2.50×10^-^**^7^** |  | 0.96 | 0% |
| rs130067 | 6p21 | A/C | 31118511 | *CCHCR1* | Genotyped | 1.08 (0.89-1.30) | 0.45 |  | 1.07 (1.04-1.10) | 7.13×10^-^**^5^** |  | 0.94 | 0% |
| rs3096702 | 6p21 | C/T | 32192331 | *NOTCH4* | Genotyped | 1.11 (0.88-1.39) | 0.4 |  | 1.07 (1.04-1.10) | 4.54×10^-^**^6^** |  | 0.76 | 0% |
| rs115306967 | 6p21 | G/C | 32400939 | *HLA-DRB6* | Imputed | 0.89 (0.71-1.10) | 0.27 |  | 0.93 (0.90-0.96) | 2.29×10^-^**^7^** |  | 0.68 | 0% |
| rs9443189 | 6q14 | A/G | 76495882 | *MYO6* | Imputed | 0.91 (0.76-1.08) | 0.27 |  | 0.94 (0.90-0.97) | 8.08×10^-^**^4^** |  | 0.72 | 0% |
| rs2273669 | 6q21 | A/G | 109285189 | *ARMC2* | Genotyped | 1.19 (0.85-1.65) | 0.32 |  | 1.08 (1.04-1.12) | 6.62×10^-^**^5^** |  | 0.58 | 0% |
| rs1933488 | 6q25 | G/A | 153441079 | *RGS17* | Genotyped | 1.24 (0.99-1.56) | 0.06 |  | 1.12 (1.09-1.14) | 3.34×10^-^**^15^** |  | 0.37 | 0% |
| rs651164 | 6q25 | A/G | 160581374 | *SLC22A1* | Genotyped | 1.09 (0.92-1.30) | 0.3 |  | 1.14 (1.11-1.17) | 3.63×10^-^**^19^** |  | 0.61 | 0% |
| rs9364554 | 6q25 | C/T | 160833664 | *SLC22A3* | Genotyped | 1.03 (0.86-1.22) | 0.82 |  | 1.10 (1.07-1.13) | 6.37×10^-^**^11^** |  | 0.43 | 0% |
| rs12155172 | 7p15 | G/A | 20994491 |  | Genotyped | 1.02 (0.84-1.22) | 0.9 |  | 1.10 (1.07-1.13) | 2.77×10^-^**^9^** |  | 0.4 | 0% |
| rs10486567 | 7p15 | A/G | 27976563 | *JAZF1* | Genotyped | 0.96 (0.75-1.22) | 0.75 |  | 1.15 (1.11-1.18) | 2.49×10^-16^ |  | 0.15 | 50.6% |
| rs56232506 | 7p12 | G/A | 47437244 | *TNS3* | Imputed | 1.20 (1.00-1.44) | 0.06 |  | 1.07 (1.04-1.09) | 2.06×10^-6^ |  | 0.23 | 32% |
| rs6465657 | 7q21 | C/T | 97816327 | *LMTK2* | Genotyped | 0.99 (0.78-1.26) | 0.97 |  | 0.90 (0.87-0.92) | 2.71×10^-^**^15^** |  | 0.43 | 0% |
| rs2928679 | 8p21 | C/T | 23438975 | *SLC25A37* | Genotyped | 1.26 (0.98-1.62) | 0.08 |  | 1.05 (1.03-1.08) | 1.98×10^-4^ |  | 0.17 | 46.9% |
| rs1512268 | 8p21 | G/A | 23526463 | *NKX3.1* | Genotyped | 1.11 (0.93-1.34) | 0.27 |  | 1.14 (1.10-1.18) | 3.35×10^-^**^20^** |  | 0.82 | 0% |
| rs11135910 | 8p21 | C/T | 25892142 | *EBF2* | Genotyped | 1.30 (0.81-2.09) | 0.29 |  | 1.11 (1.07-1.14) | 1.38×10^-^**^7^** |  | 0.5 | 0% |
| rs12543663 | 8q24 | A/C | 127924659 |  | Genotyped | 1.05 (0.72-1.54) | 0.79 |  | 1.14 (1.11-1.17) | 5.09×10^-^**^19^** |  | 0.67 | 0% |
| rs10086908 | 8q24 (5) | T/C | 128011937 | *PCAT1* | Genotyped | 0.86 (0.68-1.08) | 0.2 |  | 0.87 (0.84-0.90) | 6.99×10^-^**^20^** |  | 0.9 | 0% |
| *rs16901979* | *8q24 (2)* | C/A | *128124916* | *PRNCR1* | Genotyped | *1.43 (1.19-1.72)* | *1.31×10^-4^* |  | *1.66 (1.59-1.74)* | *1.05*×*10^-45^* |  | *0.14* | *54.7%* |
| rs188140481 | 8q24 | T**^f^** | 128191672 | *CASC19* | Imputed |  |  |  |  |  |  |  |  |
| **rs16902094** | **8q24** | **A/G** | **128320346** | *CASC8*/*CASC21* | **Genotyped** | **0.97 (0.80-1.18)** | **0.8** |  | **1.21 (1.17-1.24)** | **3.03×10^-23^** |  | **0.03** | **77.6%** |
| rs445114 | 8q24 | T/C | 128323181 | *CASC8*/*CASC21* | Genotyped | 0.89 (0.75-1.06) | 0.18 |  | 0.87 (0.84-0.90) | 5.74×10^-^**^21^** |  | 0.85 | 0% |
| rs620861 | 8q24 (4) | C/T | 128335673 | *CASC8*/*CASC21* | Genotyped | 0.93 (0.79-1.11) | 0.46 |  | 0.87 (0.84-0.90) | 4.08×10^-^**^22^** |  | 0.41 | 0% |
| *rs6983267* | *8q24 (3)* | T/G | *128413305* | *CASC8* | Genotyped | *1.36 (1.14-1.61)* | *6.04×10^-4^* |  | *1.25 (1.22-1.28)* | *2.03*×*10^-57^* |  | *0.39* | *0%* |
| *rs4242382* | *8q24 (1)* | G/A | *128517573* |  | Genotyped | *1.65 (1.31-2.07)* | *2.01×10^-5^* |  | *1.44 (1.40-1.48)* | *6.61*×*10^-68^* |  | *0.25* | *26%* |
| rs17694493 | 9p21 | C/G | 22041998 | *CDKN2B-AS1* | Genotyped | 0.78 (0.43-1.42) | 0.42 |  | 1.08 (1.04-1.12) | 9.44×10^-5^ |  | 0.29 | 10.8% |
| rs1571801 | 9q33 | C/A | 124427373 | *DAB21P* | Genotyped | 1.15 (0.76-1.75) | 0.52 |  | 1.04 (1.01-1.08) | 4.61×10^-^**^3^** |  | 0.64 | 0% |
| rs76934034 | 10q11 | T**^f^** | 46082985 | *MARCH8* | Imputed |  |  |  |  |  |  |  |  |
| *rs10993994* | *10q11* | C/T | *51549496* | *MSMB* | Genotyped | *1.25 (1.05-1.48)* | *0.01* |  | *1.23 (1.20-1.26)* | *7.25*×*10^-49^* |  | *0.89* | *0%* |
| rs3850699 | 10q24 | T/C | 104414221 | *TRIM8* | Genotyped | 1.00 (0.78-1.29) | 0.98 |  | 0.92 (0.89-0.95) | 2.96×10^-^**^8^** |  | 0.5 | 0% |
| rs4962416 | 10q26 | T/C | 126696872 | *CTBP2* | Imputed**^g^** | 1.05 (0.41-2.68) | 0.92 |  | 1.04 (1.01-1.07) | 5.75×10^-^**^3^** |  | 0.99 | 0% |
| rs7127900 | 11p15 | G/A | 2233574 |  | Genotyped | 1.08 (0.82-1.41) | 0.61 |  | 1.23 (1.20-1.27) | 5.60×10^-^**^35^** |  | 0.33 | 0% |
| rs12418451 | 11q13 | G/A | 68935419 |  | Genotyped | 0.92 (0.65-1.29) | 0.64 |  | 1.14 (1.11-1.17) | 6.41×10^-18^ |  | 0.22 | 34.6% |
| rs11228565 | 11q13 | G/A | 68978580 |  | Genotyped | 0.93 (0.63-1.36) | 0.71 |  | 1.23 (1.20-1.26) | 1.04×10^-34^ |  | 0.15 | 51.2% |
| **rs7931342** | **11q13** | **T/G** | **68994497** |  | **Genotyped** | **0.94 (0.77-1.14)** | **0.55** |  | **1.19 (1.17-1.22)** | **1.54×10^-37^** |  | **0.02** | **82.3%** |
| rs10896449 | 11q13 | A/G | 68994667 |  | Genotyped | 1.01 (0.74-1.38) | 0.97 |  | 1.20 (1.17-1.23) | 2.90×10^-39^ |  | 0.29 | 11.6% |
| rs11568818 | 11q22 | A/G | 102401661 | *MMP7* | Genotyped | 1.01 (0.74-1.37) | 0.95 |  | 0.90 (0.87-0.93) | 1.77×10^-^**^13^** |  | 0.47 | 0% |
| rs11214775 | 11q23 | G/A | 113807181 | *HTR3B* | Imputed | 1.05 (0.75-1.47) | 0.79 |  | 0.92 (0.87-0.95) | 3.00×10^-7^ |  | 0.45 | 0% |
| rs80130819 | 12q13 | A**^f^** | 48419618 | *RP1-228P16.4* | Imputed |  |  |  |  |  |  |  |  |
| rs10875943 | 12q13 | C/T | 49676010 | *TUBA1C*/*PRPH* | Genotyped | 0.80 (0.63-1.04) | 0.09 |  | 0.91 (0.88-0.94) | 1.54×10^-^**^10^** |  | 0.35 | 0% |
| rs902774 | 12q13 | G/A | 53273904 | *KRT8* | Imputed**^g^** | 2.45 (0.82-7.37) | 0.11 |  | 1.12 (1.08-1.15) | 1.09×10^-9^ |  | 0.16 | 48.9% |
| rs1270884 | 12q24 | C/T | 114685571 |  | Genotyped | 0.94 (0.78-1.14) | 0.54 |  | 1.08 (1.05-1.10) | 1.63×10^-7^ |  | 0.17 | 47.4% |
| rs8008270 | 14q22 | C**^f^** | 53372330 | *FERMT2* | Imputed |  |  |  |  |  |  |  |  |
| *rs7153648* | *14q23* | *G/C* | *61122526* | *SIX1* | Imputed *(MAF<0.01)* | *1.35 (1.00-1.80)* | *0.05* |  | *1.13 (1.06-1.19)* | 1.36×10^-^**^4^** |  | *0.24* | *26.7%* |
| rs7141529 | 14q24 | C/T | 69126744 | *RAD51B* | Genotyped | 0.85 (0.68-1.06) | 0.15 |  | 0.92 (0.90-0.95) | 1.40×10^-^**^8^** |  | 0.44 | 0% |
| rs8014671 | 14q24 |  | 71092256 | *TTC9* | N/A**^e^** |  |  |  |  |  |  |  |  |
| rs12051443 | 16q22 | A/G | 71691329 | *PHLPP2* | Imputed | 0.90 (0.73-1.10) | 0.3 |  | 0.95 (0.92-0.98) | 2.69×10^-^**^4^** |  | 0.59 | 0% |
| rs684232 | 17p13 | G/A | 618965 | *VPS53* | Genotyped | 0.88 (0.74-1.04) | 0.14 |  | 0.91 (0.88-0.94) | 1.85×10^-^**^11^** |  | 0.68 | 0% |
| rs11649743 | 17q12 | G/A | 36074979 | *HNF1B* | Genotyped | 0.94 (0.79-1.12) | 0.5 |  | 0.89 (0.85-0.92) | 2.56×10^-^**^11^** |  | 0.55 | 0% |
| *rs4430796* | *17q12* | A/G | *36098040* | *HNF1B* | Imputed | *0.73 (0.60-0.90)* | *3.34×10^-3^* |  | *0.81 (0.79-0.82)* | *0* |  | *0.36* | *0%* |
| rs138213197 | 17q12 | C**^f^** | 46805705 | *HOXB13* | Imputed |  |  |  |  |  |  |  |  |
| rs11650494 | 17q21 | G/A | 47345186 | *HOXB13* | Imputed**^g^** | 1.67 (0.68-4.06) | 0.26 |  | 1.14 (1.09-1.19) | 1.56×10^-7^ |  | 0.41 | 0% |
| rs1859962 | 17q24 | T/G | 69108753 | *CASC17* | Genotyped | 1.05 (0.88-1.25) | 0.59 |  | 1.19 (1.16-1.22) | 5.46×10^-36^ |  | 0.17 | 45.8% |
| rs7241993 | 18q23 | C/T | 76773973 | *SALL3* | Genotyped | 1.00 (0.85-1.19) | 0.97 |  | 0.93 (0.90-0.96) | 6.62×10^-7^ |  | 0.38 | 0% |
| rs8102476 | 19q13 | T/C | 3873561 | *PPP1R14A* | Genotyped | 1.05 (0.88-1.25) | 0.65 |  | 1.08 (1.05-1.10) | 1.45×10^-7^ |  | 0.75 | 0% |
| rs11672691 | 19q13 | G/A | 41985587 | *PCAT19* | Genotyped | 1.03 (0.86-1.22) | 0.77 |  | 0.90 (0.87-0.93) | 2.36×10^-11^ |  | 0.18 | 44% |
| rs887391 | 19q13 | T/C | 41985624 | *PCAT19* | Genotyped | 1.05 (0.88-1.25) | 0.64 |  | 0.91 (0.94-0.88) | 6.21×10^-9^ |  | 0.1 | 64% |
| **rs2735839** | **19q13** | **G/A** | **51364623** | ***KLK3*** | **Genotyped** | **1.04 (0.87-1.24)** | **0.74** |  | **0.84 (0.80-0.88)** | **1.26×10^-16^** |  | **0.03** | **79.5%** |
| rs12480328 | 20q13 | T/C | 49527922 | *ADNP* | Imputed | 0.83 (0.56-1.16) | 0.27 |  | 0.89 (0.84-0.95) | 3.77×10^-5^ |  | 0.65 | 0% |
| **rs2427345** | **20q13** | **C/T** | **61015611** | *RBBP8NL* | **Genotyped** | **1.22 (0.97-1.53)** | **0.09** |  | **0.93 (0.91-0.96)** | **1.52×10^-6^** |  | **0.02** | **81.2%** |
| *rs6062509* | *20q13* | G/T | *62362563* | *ZGPAT* | Genotyped | *1.24 (1.03-1.48)* | *0.02* |  | *1.12 (1.09-1.14)* | *3.90*×*10^-13^* |  | *0.27* | *17.1%* |
| rs1041449 | 21q22 | A/G | 42901421 | *TMPRSS2* | Genotyped | 1.14 (0.92-1.42) | 0.24 |  | 1.07 (1.04-1.10) | 1.13×10^-6^ |  | 0.58 | 0% |
| rs2238776 | 22q11 | G/A | 19757892 | *TBX1* | Imputed | 0.91 (0.75-1.10) | 0.33 |  | 0.93 (0.90-0.97) | 2.99×10^-^**^4^** |  | 0.77 | 0% |
| rs9623117 | 22q13 | T/C | 40452119 | *TNRC6B* | Genotyped | 0.89 (0.55-1.43) | 0.64 |  | 1.08 (1.04-1.11) | 8.18×10^-^**^6^** |  | 0.42 | 0% |
| **rs5759167** | **22q13** | **G/T** | **43500212** | ***BIL*/*TTLL1*** | **Imputed** | **1.11 (0.91-1.35)** | **0.29** |  | **0.87 (0.85-0.88)** | **0** |  | **0.01** | **84.1%** |
| rs2405942 | Xp22 | A/G | 9814135 | *SHROOM2* | Genotyped | 0.84 (0.55-1.26) | 0.41 |  | 0.93 (0.91-0.96) | 2.14×10^-^**^8^** |  | 0.6 | 0% |
| rs5945572 | Xp11 | G/A | 51229683 | *NUDT10*/*NUDT11* | Genotyped | 1.10 (0.70-1.72) | 0.72 |  | 1.11 (1.09-1.13) | 5.67×10^-^**^24^** |  | 0.97 | 0% |
| rs5945619 | Xp11 | T/C | 51241672 | *NUDT11* | Genotyped | 1.08 (0.69-1.68) | 0.78 |  | 1.11 (1.09-1.13) | 2.61×10^-^**^24^** |  | 0.9 | 0% |
| rs2807031 | Xp11 | T/C | 52896949 | *XAGE3* | Imputed | 1.26 (0.59-2.71) | 0.55 |  |  |  |  |  |  |
| rs5919432 | Xq12 | T**^f^** | 67021550 | *AR* | Imputed |  |  |  |  |  |  |  |  |
| rs6625711 | Xq13 |  | 70139850 | *SLC7A* | N/A**^e^** |  |  |  |  |  |  |  |  |
| rs4844289 | Xq13 | G/A | 70407983 | *NLGN3-BCYRN1* | Imputed | 1.07 (0.82-1.40) | 0.61 |  |  |  |  |  |  |
| Loci identified in populations of Japanese ancestry | | | | |  |  |  |  |  |  |  |  |  |
| rs13385191 | 2p24 | A/G | 20888265 | *C2orf43* | Imputed | 0.97 (0.82-1.16) | 0.77 |  | 1.05 (1.02–1.08) | 3.01×10^-3^ |  | 0.41 | 0% |
| rs2028898 | 2p11 | G/A | 85777270 | *GGCX* | Imputed | 0.89 (0.73-1.05) | 0.16 |  | 0.93 (0.90–0.96) | 1.97×10^-^**^6^** |  | 0.55 | 0% |
| rs2055109 | 3p11 | T/C | 87467332 |  | Imputed | 1.21 (0.88-1.67) | 0.23 |  | 1.01 (1.00–1.02) | 0.02 |  | 0.26 | 20.6% |
| *rs12653946* | *5p15* | C/T | *1895829* | *IRX4* | Genotyped | *1.24 (1.04-1.48)* | *0.02* |  | *1.08 (1.06–1.11)* | *9.1×10^-9^* |  | *0.14* | *53.7%* |
| *rs1983891* | *6p21* | C/T | *41536427* | *FOXP4* | Imputed | *1.22 (1.02-1.47)* | *0.03* |  | *1.07 (1.04–1.10)* | *3.54×10^-6^* |  | *0.17* | *46.6%* |
| *rs339331* | *6q22* | T/C | *117210052* | *RFX6* | Imputed | *0.76 (0.62-0.94)* | *9.71×10^-3^* |  | *0.93 (0.90–0.96)* | *3.61*×*10^-7^* |  | *0.07* | *70.2%* |
| rs2252004 | 10q26 | C/A | 122844709 |  | Imputed | 0.91 (0.73-1.15) | 0.45 |  | 0.98 (0.94–1.03) | 0.43 |  | 0.56 | 0% |
| rs1938781 | 11q12 |  | 58915110 | *FAM111A* | N/A**^e^** |  |  |  |  |  |  |  |  |
| rs9600079 | 13q22 |  | 73728139 |  | N/A**^e^** |  |  |  |  |  |  |  |  |

**^a^**Major/minor allele based on the frequencies in iCOGS data from Chinese population.

^b^Odds ratio values with 95% confidence intervals calculated for minor alleles in iCOGS data from Chinese population.

^c^*P* value for Cochran’s Q statistic estimated using iCOGS data sets from Chinese and European descendants.

^d^Index of heterogeneity estimated between iCOGS data sets from Chinese and European descendants.

^e^Low imputation certainty (posterior probability < 0.9).

^f^Only one allele observed in iCOGS data from Chinese population.

^g^MAF observed in iCOGS data from Chinese population less than 1% (rs4962416[C] = 0.007; rs902774[A] = 0.007; rs11650494 [A] = 0.009).

NOTE: SNPs with significant association with prostate cancer (*P*<0.05) in iCOGS data from Chinese population are in italics. SNPs with an evidence of heterogeneity (*P*_het_<0.05) between iCOGS data derived from Chinese and European populations are in bold.

**Supplementary Table S4. Meta-analysis of Chinese iCOGS and Chinese GWAS data using SNPs associated with prostate cancer with *P*<0.001 in Chinese iCOGS array data**.

|  | |  |  |  | **Chinese iCOGS** | | | |  | **Chinese GWAS** | | | |  | **Combined** | |
| --- | --- | --- | --- | --- | --- | --- | --- | --- | --- | --- | --- | --- | --- | --- | --- | --- |
| **SNP** | **Location** | | **Alleles^a^** | **Reported**  **SNP** | **MAF** | | **OR (95% CI)** | ***P* < 1×10^-3^** |  | **MAF** | | **OR (95% CI)** | ***P*** |  | **OR (95% CI)** | ***P*** |
|  |  |  |  |  | **Case** | **Control** |  |  |  | **Case** | **Control** |  |  |  |  |  |
| **rs2173049** | | 1p36 | C/T |  | 0.163 | 0.113 | 1.53 (1.19-1.97) | 9.76×10^-4^ |  | 0.142 | 0.143 | 0.98 (0.83-1.15) | 8.55×10^-1^ |  | 1.12 (0.97-1.28) | 1.11×10^-1^ |
| **rs4354529** | | 1p36 | A/G |  | 0.084 | 0.047 | 1.85 (1.29-2.64) | 7.62×10^-4^ |  | 0.062 | 0.064 | 0.97 (0.77-1.23) | 8.29×10^-1^ |  | 1.18 (0.97-1.43) | 9.98×10^-2^ |
| **rs6701216** | | 1q21 | C/T |  | 0.380 | 0.309 | 1.37 (1.15-1.65) | 6.34×10^-4^ |  | 0.339 | 0.363 | 0.90 (0.80-1.01) | 8.87×10^-2^ |  | 1.02 (0.93-1.13) | 6.56×10^-1^ |
| **rs1864346** | | 1q23 | C/T |  | 0.487 | 0.410 | 1.38 (1.16-1.64) | 3.73×10^-4^ |  | 0.448 | 0.441 | 1.03 (0.92-1.16) | 6.33×10^-1^ |  | 1.12 (1.02-1.24) | **1.77×10^-2^** |
| **rs2494262** | | 1q23 | A/C |  | 0.228 | 0.303 | 0.68 (0.56-0.83) | 1.26×10^-4^ |  | 0.280 | 0.290 | 0.95 (0.84-1.08) | 4.56×10^-1^ |  | 0.86 (0.77-0.96) | **6.21×10^-3^** |
| **rs2511214** | | 1q23 | T/G |  | 0.228 | 0.303 | 0.68 (0.56-0.83) | 1.26×10^-4^ |  | 0.287 | 0.304 | 0.92 (0.81-1.05) | 2.39×10^-1^ |  | 0.84 (0.75-0.94) | **1.58×10^-3^** |
| **rs12127203** | | 1q25 | A/G |  | 0.240 | 0.178 | 1.45 (1.18-1.80) | 5.90×10^-4^ |  | 0.173 | 0.159 | 1.11 (0.95-1.30) | 2.10×10^-1^ |  | 1.22 (1.08-1.39) | **1.99×10^-3^** |
| **rs12567052** | | 1q32 | G/A |  | 0.241 | 0.321 | 0.67 (0.55-0.81) | 5.89×10^-5^ |  | 0.285 | 0.291 | 0.97 (0.86-1.10) | 6.57×10^-1^ |  | 0.87 (0.78-0.97) | **9.92×10^-3^** |
| **rs1415991** | | 1q41 | A/G |  | 0.121 | 0.178 | 0.63 (0.49-0.81) | 3.40×10^-4^ |  | 0.147 | 0.151 | 0.97 (0.83-1.14) | 6.68×10^-1^ |  | 0.86 (0.75-0.98) | **2.45×10^-2^** |
| **rs4854379** | | 2p25 | C/T |  | 0.349 | 0.422 | 0.74 (0.62-0.89) | 9.27×10^-4^ |  | 0.386 | 0.382 | 1.02 (0.91-1.15) | 7.42×10^-1^ |  | 0.92 (0.84-1.02) | 1.15×10^-1^ |
| **rs6758730** | | 2p25 | A/G |  | 0.189 | 0.251 | 0.69 (0.55-0.85) | 6.45×10^-4^ |  | 0.239 | 0.243 | 0.97 (0.85-1.11) | 6.99×10^-1^ |  | 0.88 (0.79-0.99) | **2.89×10^-2^** |
| **rs4670047** | | 2p24 | C/T |  | 0.407 | 0.331 | 1.38 (1.15-1.64) | 4.19×10^-4^ |  | 0.363 | 0.364 | 1.00 (0.89-1.13) | 9.63×10^-1^ |  | 1.10 (1.00-1.22) | **4.84×10^-2^** |
| **rs6755308** | | 2p21 | G/A |  | 0.053 | 0.024 | 2.30 (1.43-3.69) | 6.04×10^-4^ |  | 0.036 | 0.032 | 1.13 (0.82-1.55) | 4.36×10^-1^ |  | 1.41 (1.08-1.83) | **1.10×10^-2^** |
| **rs7569428** | | 2p12 | A/G |  | 0.377 | 0.449 | 0.74 (0.62-0.88) | 8.16×10^-4^ |  | 0.428 | 0.414 | 1.06 (0.94-1.19) | 3.26×10^-1^ |  | 0.95 (0.86-1.05) | 3.19×10^-1^ |
| **rs4539815** | | 2q12 | T/C |  | 0.281 | 0.216 | 1.42 (1.17-1.74) | 5.50×10^-4^ |  | 0.247 | 0.224 | 1.14 (1.00-1.31) | 6.45×10^-2^ |  | 1.22 (1.09-1.37) | **4.43×10^-4^** |
| **rs4849821** | | 2q14 | T/C |  | 0.265 | 0.331 | 0.72 (0.59-0.88) | 9.88×10^-4^ |  | 0.293 | 0.306 | 0.94 (0.83-1.06) | 3.14×10^-1^ |  | 0.87 (0.78-0.97) | **9.15×10^-3^** |
| **rs4849826** | | 2q14 | A/G |  | 0.265 | 0.332 | 0.71 (0.59-0.87) | 7.17×10^-4^ |  | 0.293 | 0.307 | 0.94 (0.83-1.06) | 3.20×10^-1^ |  | 0.87 (0.78-0.96) | **7.96×10^-3^** |
| **rs4849838** | | 2q14 | A/G |  | 0.281 | 0.352 | 0.71 (0.59-0.86) | 4.57×10^-4^ |  | 0.295 | 0.310 | 0.93 (0.81-1.06) | 3.15×10^-1^ |  | 0.85 (0.76-0.95) | **3.80×10^-3^** |
| **rs12617045** | | 2q24 | A/G |  | 0.201 | 0.145 | 1.49 (1.19-1.87) | 6.78×10^-4^ |  | 0.178 | 0.172 | 1.05 (0.90-1.22) | 5.30×10^-1^ |  | 1.17 (1.03-1.32) | **1.63×10^-2^** |
| **rs774516** | | 2q24 | T/C |  | 0.201 | 0.142 | 1.54 (1.22-1.95) | 2.74×10^-4^ |  | 0.177 | 0.172 | 1.04 (0.89-1.21) | 6.19×10^-1^ |  | 1.17 (1.03-1.33) | **1.59×10^-2^** |
| **rs3108779** | | 2q31 | T/G |  | 0.210 | 0.272 | 0.70 (0.57-0.86) | 8.53×10^-4^ |  | 0.248 | 0.260 | 0.94 (0.82-1.07) | 3.63×10^-1^ |  | 0.87 (0.77-0.97) | **1.03×10^-2^** |
| **rs4405725** | | 2q31 | T/A | rs12621278 | 0.231 | 0.298 | 0.70 (0.58-0.86) | 5.40×10^-4^ |  | 0.250 | 0.274 | 0.88 (0.77-1.00) | 5.74×10^-2^ |  | 0.82 (0.74-0.92) | **4.51×10^-4^** |
| **rs3762618** | | 2q31 | G/C | rs12621278 | 0.231 | 0.296 | 0.71 (0.58-0.86) | 7.13×10^-4^ |  | 0.250 | 0.274 | 0.88 (0.77-1.00) | 5.74×10^-2^ |  | 0.82 (0.74-0.92) | **5.28×10^-4^** |
| **rs10207640** | | 2q31 | A/G | rs12621278 | 0.231 | 0.298 | 0.70 (0.58-0.86) | 5.40×10^-4^ |  | 0.251 | 0.276 | 0.88 (0.77-1.00) | **4.69×10^-2^** |  | 0.82 (0.74-0.92) | **4.45×10^-4^** |
| **rs10207654** | | 2q31 | A/G | rs12621278 | 0.231 | 0.298 | 0.70 (0.58-0.86) | 5.40×10^-4^ |  | 0.251 | 0.277 | 0.87 (0.76-0.99) | **4.10×10^-2^** |  | 0.82 (0.73-0.91) | **2.54×10^-4^** |
| **rs13410475** | | 2q31 | C/A | rs12621278 | 0.230 | 0.296 | 0.70 (0.58-0.86) | 5.81×10^-4^ |  | 0.251 | 0.276 | 0.88 (0.77-1.00) | **4.69×10^-2^** |  | 0.82 (0.74-0.92) | **4.67×10^-4^** |
| **rs12621278^b^** | | 2q31 | A/G | rs12621278 | 0.231 | 0.296 | 0.71 (0.58-0.87) | 8.31×10^-4^ |  | 0.251 | 0.276 | 0.88 (0.77-1.00) | **4.69×10^-2^** |  | 0.83 (0.74-0.92) | **5.62×10^-4^** |
| **rs1574259** | | 2q31 | C/T | rs12621278 | 0.231 | 0.295 | 0.71 (0.58-0.87) | 9.86×10^-4^ |  | 0.251 | 0.276 | 0.88 (0.77-1.00) | **4.69×10^-2^** |  | 0.83 (0.74-0.92) | **6.24×10^-4^** |
| **rs1574256** | | 2q31 | C/G | rs12621278 | 0.230 | 0.296 | 0.71 (0.58-0.86) | 7.13×10^-4^ |  | 0.251 | 0.276 | 0.88 (0.77-1.00) | **4.69×10^-2^** |  | 0.83 (0.74-0.92) | **5.21×10^-4^** |
| **rs10210544** | | 2q31 | A/G | rs12621278 | 0.263 | 0.330 | 0.72 (0.59-0.87) | 8.75×10^-4^ |  | 0.288 | 0.302 | 0.93 (0.82-1.05) | 2.95×10^-1^ |  | 0.86 (0.78-0.96) | **5.54×10^-3^** |
| **rs12052253** | | 2q31 | A/T | rs12621278 | 0.231 | 0.296 | 0.71 (0.58-0.87) | 8.31×10^-4^ |  | 0.251 | 0.276 | 0.88 (0.77-1.00) | **4.73×10^-2^** |  | 0.83 (0.74-0.92) | **5.63×10^-4^** |
| **rs16860426** | | 2q31 | A/T | rs12621278 | 0.228 | 0.295 | 0.70 (0.57-0.86) | 5.23×10^-4^ |  | 0.250 | 0.277 | 0.87 (0.76-0.99) | **3.85×10^-2^** |  | 0.82 (0.73-0.91) | **2.53×10^-4^** |
| **rs7564416** | | 2q31 | G/A | rs12621278 | 0.246 | 0.310 | 0.72 (0.59-0.87) | 9.05×10^-4^ |  | 0.267 | 0.289 | 0.90 (0.79-1.02) | 9.85×10^-2^ |  | 0.84 (0.75-0.94) | **1.57×10^-3^** |
| **rs12622816** | | 2q31 | G/A | rs12621278 | 0.265 | 0.331 | 0.72 (0.59-0.87) | 8.92×10^-4^ |  | 0.276 | 0.307 | 0.86 (0.76-0.98) | **1.94×10^-2^** |  | 0.82 (0.73-0.91) | **1.56×10^-4^** |
| **rs1076594** | | 2q31 | A/G | rs12621278 | 0.295 | 0.364 | 0.72 (0.60-0.87) | 6.77×10^-4^ |  | 0.310 | 0.330 | 0.91 (0.81-1.03) | 1.51×10^-1^ |  | 0.85 (0.77-0.94) | **1.81×10^-3^** |
| **rs1076596** | | 2q31 | C/T | rs12621278 | 0.265 | 0.332 | 0.71 (0.59-0.86) | 6.58×10^-4^ |  | 0.275 | 0.304 | 0.87 (0.77-0.99) | **2.87×10^-2^** |  | 0.82 (0.74-0.91) | **2.43×10^-4^** |
| **rs6759557** | | 2q31 | G/A |  | 0.372 | 0.445 | 0.73 (0.61-0.87) | 6.15×10^-4^ |  | 0.398 | 0.405 | 0.97 (0.86-1.09) | 6.26×10^-1^ |  | 0.89 (0.81-0.98) | **2.14×10^-2^** |
| **rs1040336** | | 3p14 | C/T |  | 0.455 | 0.529 | 0.74 (0.62-0.88) | 6.89×10^-4^ |  | 0.491 | 0.501 | 0.96 (0.86-1.08) | 5.00×10^-1^ |  | 0.89 (0.81-0.98) | **1.42×10^-2^** |
| **rs12493170** | | 3p14 | A/G |  | 0.457 | 0.531 | 0.74 (0.62-0.88) | 7.57×10^-4^ |  | 0.494 | 0.500 | 0.97 (0.86-1.09) | 6.61×10^-1^ |  | 0.89 (0.81-0.98) | **2.19×10^-2^** |
| **rs7623262** | | 3p12 | A/G |  | 0.310 | 0.381 | 0.72 (0.59-0.86) | 5.07×10^-4^ |  | 0.368 | 0.370 | 0.99 (0.88-1.11) | 8.69×10^-1^ |  | 0.90 (0.82-1.00) | **4.56×10^-2^** |
| **rs13319291** | | 3q22 | G/A |  | 0.401 | 0.484 | 0.71 (0.59-0.84) | 1.37×10^-4^ |  | 0.450 | 0.477 | 0.90 (0.80-1.02) | 8.40×10^-2^ |  | 0.83 (0.76-0.92) | **3.64×10^-4^** |
| **rs6531601** | | 4p14 | A/T |  | 0.320 | 0.252 | 1.39 (1.15-1.67) | 7.44×10^-4^ |  | 0.274 | 0.254 | 1.11 (0.97-1.27) | 1.39×10^-1^ |  | 1.20 (1.07-1.34) | **1.38×10^-3^** |
| **rs11940785** | | 4q21 | C/T |  | 0.371 | 0.297 | 1.39 (1.16-1.66) | 4.50×10^-4^ |  | 0.321 | 0.311 | 1.05 (0.93-1.19) | 4.83×10^-1^ |  | 1.15 (1.04-1.27) | **8.65×10^-3^** |
| **rs1354101** | | 4q21 | T/C |  | 0.356 | 0.283 | 1.39 (1.16-1.67) | 4.23×10^-4^ |  | 0.310 | 0.298 | 1.06 (0.93-1.21) | 4.27×10^-1^ |  | 1.17 (1.05-1.30) | **5.38×10^-3^** |
| **rs600121** | | 4q28 | G/A |  | 0.515 | 0.430 | 1.39 (1.17-1.65) | 2.01×10^-4^ |  | 0.472 | 0.471 | 1.00 (0.89-1.12) | 9.45×10^-1^ |  | 1.11 (1.01-1.22) | **3.94×10^-2^** |
| **rs12513208** | | 4q28 | C/T |  | 0.350 | 0.419 | 0.74 (0.61-0.88) | 9.59×10^-4^ |  | 0.385 | 0.404 | 0.92 (0.82-1.03) | 1.81×10^-1^ |  | 0.86 (0.78-0.95) | **2.97×10^-3^** |
| **rs11721827** | | 4q35 | A/C |  | 0.179 | 0.244 | 0.70 (0.57-0.86) | 6.66×10^-4^ |  | 0.217 | 0.209 | 1.05 (0.91-1.21) | 5.12×10^-1^ |  | 0.92 (0.82-1.04) | 1.81×10^-1^ |
| **rs4704506** | | 5q14 | T/G |  | 0.313 | 0.245 | 1.39 (1.15-1.68) | 6.93×10^-4^ |  | 0.278 | 0.267 | 1.06 (0.93-1.21) | 3.87×10^-1^ |  | 1.16 (1.04-1.29) | **7.91×10^-3^** |
| **rs10462558** | | 5q14 | T/G |  | 0.271 | 0.209 | 1.43 (1.17-1.76) | 6.25×10^-4^ |  | 0.224 | 0.227 | 0.98 (0.85-1.12) | 7.86×10^-1^ |  | 1.10 (0.98-1.23) | 9.72×10^-2^ |
| **rs4913029** | | 5q32 | A/G |  | 0.069 | 0.111 | 0.58 (0.42-0.80) | 9.52×10^-4^ |  | 0.086 | 0.096 | 0.88 (0.72-1.07) | 2.27×10^-1^ |  | 0.78 (0.66-0.93) | **4.80×10^-3^** |
| **rs12654453** | | 5q35 | G/T |  | 0.382 | 0.460 | 0.74 (0.62-0.88) | 5.39×10^-4^ |  | 0.417 | 0.407 | 1.04 (0.92-1.18) | 5.31×10^-1^ |  | 0.92 (0.83-1.02) | 1.19×10^-1^ |
| **rs12518133** | | 5q35 | T/A |  | 0.387 | 0.463 | 0.74 (0.63-0.88) | 6.52×10^-4^ |  | 0.433 | 0.419 | 1.06 (0.94-1.19) | 3.22×10^-1^ |  | 0.95 (0.86-1.04) | 2.57×10^-1^ |
| **rs10866644** | | 5q35 | A/G |  | 0.434 | 0.521 | 0.72 (0.61-0.85) | 1.37×10^-4^ |  | 0.490 | 0.504 | 0.95 (0.85-1.06) | 3.47×10^-1^ |  | 0.87 (0.79-0.96) | **3.98×10^-3^** |
| **rs2753238** | | 6p25 | A/C |  | 0.308 | 0.240 | 1.39 (1.15-1.68) | 7.02×10^-4^ |  | 0.282 | 0.271 | 1.06 (0.93-1.20) | 4.00×10^-1^ |  | 1.15 (1.04-1.28) | **8.36×10^-3^** |
| **rs2773318** | | 6p25 | G/A |  | 0.309 | 0.242 | 1.39 (1.15-1.68) | 7.70×10^-4^ |  | 0.282 | 0.271 | 1.06 (0.93-1.20) | 4.00×10^-1^ |  | 1.15 (1.04-1.28) | **8.69×10^-3^** |
| **rs2224391** | | 6p25 | A/C |  | 0.311 | 0.243 | 1.40 (1.16-1.69) | 5.95×10^-4^ |  | 0.282 | 0.271 | 1.05 (0.92-1.19) | 4.27×10^-1^ |  | 1.15 (1.03-1.28) | **1.10×10^-2^** |
| **rs1495980** | | 6p22 | A/G |  | 0.197 | 0.142 | 1.50 (1.19-1.89) | 7.27×10^-4^ |  | 0.184 | 0.177 | 1.04 (0.90-1.21) | 5.78×10^-1^ |  | 1.16 (1.02-1.31) | **2.38×10^-2^** |
| **rs13202332** | | 6q21 | G/T |  | 0.173 | 0.234 | 0.68 (0.55-0.85) | 6.07×10^-4^ |  | 0.215 | 0.222 | 0.96 (0.84-1.10) | 5.46×10^-1^ |  | 0.87 (0.77-0.98) | **2.02×10^-2^** |
| **rs4708737** | | 6q27 | A/G |  | 0.202 | 0.268 | 0.70 (0.57-0.85) | 5.45×10^-4^ |  | 0.230 | 0.214 | 1.10 (0.96-1.26) | 1.73×10^-1^ |  | 0.95 (0.85-1.07) | 4.14×10^-1^ |
| **rs10235505** | | 7p21 | G/A |  | 0.347 | 0.266 | 1.45 (1.20-1.74) | 9.09×10^-4^ |  | 0.304 | 0.312 | 0.96 (0.85-1.09) | 5.27×10^-1^ |  | 1.09 (0.98-1.21) | 9.84×10^-2^ |
| **rs4723862** | | 7p14 | G/A |  | 0.014 | 0.042 | 0.33 (0.18-0.62) | 5.10×10^-4^ |  | 0.024 | 0.027 | 0.88 (0.61-1.27) | 4.94×10^-1^ |  | 0.69 (0.50-0.94) | **1.82×10^-2^** |
| **rs739693** | | 7q31 | C/T |  | 0.256 | 0.323 | 0.71 (0.59-0.87) | 7.44×10^-4^ |  | 0.282 | 0.286 | 0.98 (0.86-1.11) | 7.73×10^-1^ |  | 0.89 (0.80-0.99) | **3.59×10^-2^** |
| **rs6965467** | | 7q31 | T/C |  | 0.256 | 0.323 | 0.71 (0.58-0.86) | 6.39×10^-4^ |  | 0.282 | 0.284 | 0.99 (0.87-1.12) | 8.65×10^-1^ |  | 0.90 (0.81-1.00) | **4.67×10^-2^** |
| **rs337303** | | 7q31 | T/G |  | 0.424 | 0.498 | 0.73 (0.61-0.87) | 3.97×10^-4^ |  | 0.470 | 0.487 | 0.93 (0.83-1.04) | 2.31×10^-1^ |  | 0.86 (0.79-0.95) | **2.75×10^-3^**  1 |
| **rs3800562** | | 7q32 | A/G |  | 0.303 | 0.238 | 1.40 (1.15-1.71) | 7.16×10^-4^ |  | 0.263 | 0.267 | 0.98 (0.86-1.12) | 7.78×10^-1^ |  | 1.09 (0.98-1.22) | 1.06×10^-1^ |
| **rs2952635** | | 7q36 | G/A |  | 0.108 | 0.067 | 1.73 (1.26-2.37) | 6.75×10^-4^ |  | 0.066 | 0.057 | 1.17 (0.92-1.49) | 1.88×10^-1^ |  | 1.35 (1.12-1.63) | **2.03×10^-3^** |
| **rs4716858** | | 7q36 | C/T |  | 0.437 | 0.364 | 1.36 (1.14-1.62) | 6.77×10^-4^ |  | 0.398 | 0.399 | 1.00 (0.89-1.12) | 9.62×10^-1^ |  | 1.10 (1.00-1.21) | 5.97×10^-2^ |
| **rs576405** | | 8p23 | A/G |  | 0.429 | 0.492 | 0.73 (0.61-0.86) | 3.10×10^-4^ |  | 0.448 | 0.455 | 0.97 (0.86-1.09) | 6.15×10^-1^ |  | 0.89 (0.81-0.98) | **1.52×10^-2^** |
| **rs6601403** | | 8p23 | T/C |  | 0.430 | 0.513 | 0.71 (0.60-0.85) | 1.58×10^-4^ |  | 0.453 | 0.457 | 0.98 (0.87-1.10) | 7.71×10^-1^ |  | 0.89 (0.81-0.98) | **1.76×10^-2^** |
| **rs1532980** | | 8p23 | T/C |  | 0.428 | 0.514 | 0.70 (0.59-0.84) | 8.65×10^-5^ |  | 0.450 | 0.454 | 0.98 (0.87-1.10) | 7.74×10^-1^ |  | 0.89 (0.80-0.98) | **1.41×10^-2^** |
| **rs1397346** | | 8q24 | C/T |  | 0.122 | 0.180 | 0.63 (0.49-0.81) | 2.83×10^-4^ |  | 0.170 | 0.170 | 1.01 (0.87-1.18) | 9.43×10^-1^ |  | 0.89 (0.78-1.01) | 7.36×10^-2^ |
| **rs7816916** | | 8q24 | G/T |  | 0.122 | 0.181 | 0.63 (0.49-0.80) | 2.47×10^-4^ |  | 0.170 | 0.169 | 1.01 (0.87-1.18) | 9.07×10^-1^ |  | 0.89 (0.78-1.01) | 7.00×10^-2^ |
| **rs1016343** | | 8q24 (2) | C/T | rs16901979 | 0.424 | 0.350 | 1.37 (1.15-1.64) | 5.09×10^-4^ |  | 0.428 | 0.351 | 1.39 (1.24-1.56) | **5.13×10^-8^** |  | 1.39 (1.25-1.53) | **8.74×10^-11^** |
| **rs7841060** | | 8q24 (2) | T/G | rs16901979 | 0.425 | .353 | 1.36 (1.14-1.62) | 8.06×10^-4^ |  | 0.4  rs9512987  28 | 0.351 | 1.38 (1.23-1.55) | **6.65×10^-8^** |  | 1.37 (1.24-1.51) | **2.67×10^-10^** |
| **rs1456316** | | 8q24 (2) | T/A | ? | 0.292 | 0.381 | 0.67 (0.56-0.80) | 2.16×10^-5^ |  | 0.298 | 0.355 | 0.77 (0.68-0.88) | **8.87×10^-5^** |  | 0.73 (0.66-0.82) | **1.30×10^-8^** |
| **rs1456315** | | 8q24 (2) | A/G | ? | 0.204 | 0.293 | 0.61 (0.50-0.75) | 3.78×10^-6^ |  | 0.197 | 0.288 | 0.61 (0.53-0.70) | **2.76×10^-13^** |  | 0.61 (0.55-0.68) | **7.93×10^-18^** |
| **rs13254738** | | 8q24 (2) | C/A | ? | 0.217 | 0.309 | 0.62 (0.51-0.76) | 3.70×10^-6^ |  | 0.186 | 0.276 | 0.60 (0.52-0.70) | **8.26×10^-12^** |  | 0.61 (0.54-0.69) | **2.22×10^-16^** |
| **rs1073997** | | 8q24 (2) | A/C | rs16901979 | 0.336 | 0.257 | 1.45 (1.20-1.74) | 1.01×10^-4^ |  | 0.336 | 0.265 | 1.41 (1.24-1.60) | **1.07×10^-7^** |  | 1.42 (1.28-1.58) | **3.72×10^-11^** |
| **rs12682344** | | 8q24 (2) | T/G | rs16901979 | 0.334 | 0.257 | 1.42 (1.18-1.71) | 1.89×10^-4^ |  | 0.336 | 0.265 | 1.41 (1.24-1.60) | **1.07×10^-7^** |  | 1.41 (1.27-1.57) | **6.73×10^-11^** |
| **rs6983561** | | 8q24 (2) | A/C | rs16901979 | 0.335 | 0.257 | 1.43 (1.19-1.72) | 1.52×10^-4^ |  | 0.336 | 0.263 | 1.42 (1.25-1.61) | **6.11×10^-8^** |  | 1.42 (1.28-1.58) | **3.09×10^-11^** |
| **rs16901949** | | 8q24 (2) | A/C | rs16901979 | 0.342 | 0.262 | 1.44 (1.19-1.73) | 1.58×10^-4^ |  | 0.336 | 0.265 | 1.41 (1.24-1.60) | **1.07×10^-7^** |  | 1.42 (1.28-1.57) | **5.75×10^-11^** |
| **rs16901950** | | 8q24 (2) | G/A | rs16901979 | 0.352 | 0.274 | 1.42 (1.18-1.70) | 1.86×10^-4^ |  | 0.336 | 0.265 | 1.41 (1.24-1.60) | **1.07×10^-7^** |  | 1.41 (1.27-1.57) | **6.59×10^-11^** |
| **rs16901952** | | 8q24 (2) | T/C | rs16901979 | 0.334 | 0.257 | 1.42 (1.18-1.71) | 1.89×10^-4^ |  | 0.336 | 0.265 | 1.41 (1.24-1.60) | **1.07×10^-7^** |  | 1.41 (1.27-1.57) | **6.73×10^-11^** |
| **rs16901953** | | 8q24 (2) | T/C | rs16901979 | 0.335 | 0.257 | 1.43 (1.19-1.72) | 1.52×10^-4^ |  | 0.336 | 0.265 | 1.41 (1.24-1.60) | **1.07×10^-7^** |  | 1.42 (1.28-1.57) | **5.42×10^-11^** |
| **rs16901959** | | 8q24 (2) | A/G | rs16901979 | 0.334 | 0.258 | 1.42 (1.18-1.70) | 2.27×10^-4^ |  | 0.336 | 0.265 | 1.41 (1.24-1.60) | **1.07×10^-7^** |  | 1.41 (1.27-1.57) | **7.93×10^-11^** |
| **rs7826388** | | 8q24 (2) | C/T | rs16901979 | 0.334 | 0.258 | 1.42 (1.18-1.70) | 2.27×10^-4^ |  | 0.336 | 0.265 | 1.41 (1.24-1.60) | **1.07×10^-7^** |  | 1.41 (1.27-1.57) | **7.93×10^-11^** |
| **rs7830341** | | 8q24 (2) | G/A | rs16901979 | 0.334 | 0.258 | 1.42 (1.18-1.70) | 2.27×10^-4^ |  | 0.336 | 0.265 | 1.41 (1.24-1.60) | **1.07×10^-7^** |  | 1.41 (1.27-1.57) | **7.93×10^-11^** |
| **rs16901966** | | 8q24 (2) | A/G | rs16901979 | 0.334 | 0.258 | 1.42 (1.18-1.70) | 2.27×10^-4^ |  | 0.336 | 0.263 | 1.42 (1.25-1.61) | **5.63×10^-8^** |  | 1.42 (1.28-1.57) | **4.67×10^-11^** |
| **rs16901967** | | 8q24 (2) | A/G | rs16901979 | 0.334 | 0.258 | 1.42 (1.18-1.70) | 2.27×10^-4^ |  | 0.336 | 0.265 | 1.41 (1.24-1.60) | **1.07×10^-7^** |  | 1.41 (1.27-1.57) | **7.93×10^-11^** |
| **rs7001069** | | 8q24 (2) | A/G | rs16901979 | 0.334 | 0.258 | 1.42 (1.18-1.70) | 2.27×10^-4^ |  | 0.336 | 0.265 | 1.41 (1.24-1.60) | **1.07×10^-7^** |  | 1.41 (1.27-1.57) | **7.93×10^-11^** |
| **rs6988257** | | 8q24 (2) | T/C | rs16901979 | 0.334 | 0.258 | 1.42 (1.18-1.70) | 2.27×10^-4^ |  | 0.336 | 0.265 | 1.41 (1.24-1.60) | **1.07×10^-7^** |  | 1.41 (1.27-1.57) | **7.93×10^-11^** |
| **rs16901969** | | 8q24 (2) | A/C | rs16901979 | 0.334 | 0.257 | 1.43 (1.19-1.72) | 1.77×10^-4^ |  | 0.336 | 0.265 | 1.41 (1.24-1.60) | **1.07×10^-7^** |  | 1.41 (1.28-1.57) | **6.38×10^-11^** |
| **rs16901970** | | 8q24 (2) | T/G | rs16901979 | 0.334 | 0.257 | 1.42 (1.18-1.71) | 1.89×10^-4^ |  | 0.336 | 0.265 | 1.41 (1.24-1.60) | **1.07×10^-7^** |  | 1.41 (1.27-1.57) | **6.73×10^-11^** |
| **rs10453084** | | 8q24 (2) | G/A | rs16901979 | 0.334 | 0.257 | 1.43 (1.19-1.72) | 1.62×10^-4^ |  | 0.336 | 0.265 | 1.41 (1.24-1.60) | **1.07×10^-7^** |  | 1.42 (1.28-1.57) | **5.85×10^-11^** |
| **rs6987723** | | 8q24 (2) | G/A | rs16901979 | 0.334 | 0.257 | 1.43 (1.19-1.72) | 1.62×10^-4^ |  | 0.336 | 0.265 | 1.41 (1.24-1.60) | **1.07×10^-7^** |  | 1.42 (1.28-1.57) | **5.85×10^-11^** |
| **rs7824451** | | 8q24 (2) | C/G | rs16901979 | 0.335 | 0.258 | 1.43 (1.19-1.72) | 1.78×10^-4^ |  | 0.336 | 0.265 | 1.41 (1.24-1.60) | **1.07×10^-7^** |  | 1.41 (1.28-1.57) | **6.40×10^-11^** |
| **rs7844219** | | 8q24 (2) | A/G | rs16901979 | 0.336 | 0.258 | 1.43 (1.19-1.72) | 1.53×10^-4^ |  | 0.336 | 0.265 | 1.41 (1.24-1.60) | **1.07×10^-7^** |  | 1.42 (1.28-1.57) | **5.56×10^-11^** |
| **rs1551512** | | 8q24 (2) | A/C | rs16901979 | 0.336 | 0.257 | 1.43 (1.19-1.72) | 1.31×10^-4^ |  | 0.336 | 0.265 | 1.41 (1.24-1.60) | **1.07×10^-7^** |  | 1.42 (1.28-1.57) | **4.69×10^-11^** |
| **rs16901979^b^** | | 8q24 (2) | C/A | rs16901979 | 0.336 | 0.257 | 1.43 (1.19-1.72) | 1.31×10^-4^ |  | 0.336 | 0.265 | 1.41 (1.24-1.60) | **1.07×10^-7^** |  | 1.42 (1.28-1.57) | **4.69×10^-11^** |
| **rs10505483** | | 8q24 (2) | G/A | rs16901979 | 0.336 | 0.257 | 1.43 (1.19-1.72) | 1.31×10^-4^ |  | 0.335 | 0.265 | 1.40 (1.23-1.59) | **1.43×10^-7^** |  | 1.41 (1.27-1.57) | **8.89×10^-11^** |
| **rs6989838** | | 8q24 (2) | T/C | rs16901979 | 0.337 | 0.257 | 1.44 (1.20-1.73) | 1.09×10^-4^ |  | 0.336 | 0.265 | 1.41 (1.24-1.60) | **1.07×10^-7^** |  | 1.42 (1.28-1.57) | **3.97×10^-11^** |
| **rs7013255** | | 8q24 (2) | A/G | rs16901979 | 0.340 | 0.257 | 1.46 (1.22-1.76) | 5.90×10^-5^ |  | 0.336 | 0.265 | 1.41 (1.24-1.60) | **1.07×10^-7^** |  | 1.43 (1.29-1.58) | **2.28×10^-11^** |
| **rs16901984** | | 8q24 (2) | T/C | rs16901979 | 0.337 | 0.257 | 1.44 (1.20-1.73) | 1.13×10^-4^ |  | 0.336 | 0.265 | 1.41 (1.24-1.60) | **1.07×10^-7^** |  | 1.42 (1.28-1.57) | **4.06×10^-11^** |
| **rs10505477** | | 8q24 (3) | C/T | rs6983267 | 0.492 | 0.415 | 1.36 (1.15-1.62) | 4.90×10^-4^ |  | 0.463 | 0.409 | 1.25 (1.11-1.40) | **1.87×10^-4^** |  | 1.28 (1.17-1.41) | **3.51×10^-7^** |
| **rs12334317** | | 8q24 (3) | T/C | rs6983267 | 0.231 | 0.172 | 1.44 (1.16-1.78) | 8.90×10^-4^ |  | 0.197 | 0.166 | 1.23 (1.06-1.43) | **7.13×10^-3^** |  | 1.29 (1.15-1.46) | **3.53×10^-5^** |
| **rs12682374** | | 8q24 (3) | G/C | rs6983267 | 0.496 | 0.419 | 1.36 (1.14-1.61) | 5.56×10^-4^ |  | 0.468 | 0.411 | 1.26 (1.12-1.42) | **1.01×10^-4^** |  | 1.29 (1.17-1.42) | **2.49×10^-7^** |
| **rs6983267^b^** | | 8q24 (3) | T/G | rs6983267 | 0.492 | 0.417 | 1.36 (1.14-1.61) | 6.04×10^-4^ |  | 0.467 | 0.411 | 1.25 (1.11-1.40) | **1.22×10^-4^** |  | 1.28 (1.16-1.41) | **4.16×10^-7^** |
| **rs10109700** | | 8q24 (1) | G/A | rs4242382 | 0.193 | 0.138 | 1.50 (1.19-1.89) | 6.31×10^-4^ |  | 0.196 | 0.147 | 1.41 (1.21-1.65) | **1.01×10^-5^** |  | 1.44 (1.26-1.63) | **3.17×10^-8^** |
| **rs9643226** | | 8q24 (1) | G/C | rs4242382 | 0.198 | 0.143 | 1.47 (1.17-1.85) | 8.88×10^-4^ |  | 0.189 | 0.140 | 1.43 (1.22-1.68) | **1.26×10^-5^** |  | 1.44 (1.27-1.65) | **4.22×10^-8^** |
| **rs1447296** | | 8q24 (1) | C/T | rs4242382 | 0.202 | 0.144 | 1.50 (1.20-1.89) | 4.83×10^-4^ |  | 0.203 | 0.150 | 1.44 (1.23-1.68) | **3.01×10^-6^** |  | 1.46 (1.28-1.66) | **6.61×10^-9^** |
| **rs10808558** | | 8q24 (1) | G/A | rs4242382 | 0.209 | 0.143 | 1.59 (1.27-2.00) | 6.77×10^-5^ |  | 0.204 | 0.144 | 1.52 (1.30-1.78) | **1.42×10^-7^** |  | 1.54 (1.36-1.76) | **5.04×10^-11^** |
| **rs7832031** | | 8q24 (1) | G/A | rs4242382 | 0.213 | 0.144 | 1.62 (1.29-2.04) | 3.53×10^-5^ |  | 0.211 | 0.150 | 1.51 (1.30-1.76) | **9.19×10^-8^** |  | 1.54 (1.36-1.75) | **1.86×10^-11^** |
| **rs4242382^b^** | | 8q24 (1) | G/A | rs4242382 | 0.214 | 0.143 | 1.65 (1.31-2.07) | 2.01×10^-5^ |  | 0.210 | 0.150 | 1.51 (1.30-1.76) | **1.05×10^-7^** |  | 1.55 (1.37-1.76) | **1.15×10^-11^** |
| **rs4314621** | | 8q24 (1) | A/G | rs4242382 | 0.214 | 0.144 | 1.63 (1.30-2.05) | 2.68×10^-5^ |  | 0.210 | 0.150 | 1.51 (1.30-1.76) | **1.05×10^-7^** |  | 1.55 (1.36-1.76) | **1.47×10^-11^** |
| **rs7812429** | | 8q24 (1) | G/A | rs4242382 | 0.208 | 0.134 | 1.72 (1.36-2.17) | 6.12×10^-6^ |  | 0.201 | 0.139 | 1.55 (1.32-1.81) | **3.97×10^-8^** |  | 1.60 (1.40-1.82) | **1.58×10^-12^** |
| **rs7812894** | | 8q24 (1) | T/A | rs4242382 | 0.208 | 0.134 | 1.71 (1.35-2.15) | 7.79×10^-6^ |  | 0.201 | 0.139 | 1.55 (1.32-1.81) | **3.97×10^-8^** |  | 1.60 (1.40-1.82) | **1.96×10^-12^** |
| **rs7814837** | | 8q24 (1) | G/T | rs4242382 | 0.210 | 0.134 | 1.73 (1.37-2.18) | 4.61×10^-6^ |  | 0.169 | 0.120 | 1.49 (1.25-1.77) | **5.76×10^-6^** |  | 1.57 (1.37-1.80) | **1.87×10^-10^** |
| **rs7824868** | | 8q24 (1) | C/T | rs4242382 | 0.210 | 0.134 | 1.73 (1.37-2.18) | 4.61×10^-6^ |  | 0.201 | 0.140 | 1.54 (1.32-1.80) | **5.14×10^-8^** |  | 1.60 (1.40-1.82) | **2.02×10^-12^** |
| **rs13248311** | | 8q24 | C/A |  | 0.171 | 0.237 | 0.65 (0.52-0.81) | 1.60×10^-4^ |  | 0.222 | 0.201 | 1.13 (0.98-1.30) | 8.14×10^-2^ |  | 0.97 (0.86-1.09) | 5.61×10^-1^ |
| **rs13254990** | | 8q24 | C/T |  | 0.171 | 0.231 | 0.68 (0.55-0.85) | 8.05×10^-4^ |  | 0.214 | 0.194 | 1.13 (0.98-1.31) | 1.03×10^-1^ |  | 0.97 (0.86-1.10) | 6.40×10^-1^ |
| **rs13255292** | | 8q24 | C/T |  | 0.168 | 0.230 | 0.67 (0.54-0.84) | 4.56×10^-4^ |  | 0.214 | 0.191 | 1.16 (1.01-1.34) | **4.54×10^-2^** |  | 0.99 (0.88-1.12) | 8.57×10^-1^ |
| **rs1487502** | | 9p22 | C/T |  | 0.500 | 0.428 | 1.36 (1.14-1.62) | 7.90×10^-4^ |  | 0.467 | 0.460 | 1.03 (0.92-1.16) | 6.55×10^-1^ |  | 1.12 (1.02-1.23) | **2.34×10^-2^** |
| **rs263548** | | 9p22 | A/C |  | 0.436 | 0.511 | 0.74 (0.62-0.88) | 7.28×10^-4^ |  | 0.489 | 0.487 | 1.01 (0.90-1.13) | 9.26×10^-1^ |  | 0.92 (0.83-1.01) | 8.28×10^-2^ |
| **rs2515629** | | 9q31 | T/C |  | 0.031 | 0.070 | 0.44 (0.28-0.67) | 1.67×10^-4^ |  | 0.054 | 0.053 | 1.03 (0.80-1.33) | 8.36×10^-1^ |  | 0.82 (0.66-1.03) | 8.52×10^-2^ |
| **rs2274873** | | 9q31 | G/A |  | 0.029 | 0.068 | 0.42 (0.27-0.65) | 1.14×10^-4^ |  | 0.053 | 0.053 | 0.99 (0.77-1.28) | 9.39×10^-1^ |  | 0.80 (0.64-1.00) | **4.62×10^-2^** |
| **rs4837700** | | 9q33 | C/T |  | 0.453 | 0.370 | 1.41 (1.18-1.68) | 1.34×10^-4^ |  | 0.417 | 0.427 | 0.96 (0.86-1.08) | 5.03×10^-1^ |  | 1.08 (0.98-1.19) | 1.26×10^-1^ |
| **rs1335214** | | 9q33 | G/C |  | 0.467 | 0.376 | 1.46 (1.22-1.74) | 2.82×10^-5^ |  | 0.418 | 0.427 | 0.97 (0.86-1.09) | 5.75×10^-1^ |  | 1.10 (1.00-1.21) | 6.03×10^-2^ |
| **rs4837702** | | 9q33 | G/A |  | 0.456 | 0.367 | 1.45 (1.22-1.73) | 3.92×10^-5^ |  | 0.419 | 0.425 | 0.98 (0.87-1.10) | 6.77×10^-1^ |  | 1.10 (1.00-1.22) | **4.76×10^-2^** |
| **rs12238041** | | 9q33 | T/C |  | 0.464 | 0.374 | 1.45 (1.22-1.73) | 3.71×10^-5^ |  | 0.407 | 0.416 | 0.97 (0.86-1.09) | 5.49×10^-1^ |  | 1.10 (1.00-1.21) | 6.32×10^-2^ |
| **rs7040395** | | 9q33 | C/T |  | 0.445 | 0.360 | 1.43 (1.20-1.70) | 8.21×10^-5^ |  | 0.407 | 0.416 | 0.96 (0.85-1.08) | 5.37×10^-1^ |  | 1.08 (0.98-1.19) | 1.10×10^-1^ |
| **rs11253209** | | 10p15 | G/A |  | 0.055 | 0.025 | 2.28 (1.44-3.61) | 4.98×10^-4^ |  | 0.033 | 0.038 | 0.87 (0.63-1.19) | 3.86×10^-1^ |  | 1.18 (0.91-1.53) | 2.10×10^-1^ |
| **rs7095431** | | 10p14 | T/C |  | 0.255 | 0.184 | 1.49 (1.21-1.83) | 1.40×10^-4^ |  | 0.229 | 0.217 | 1.07 (0.93-1.23) | 3.15×10^-1^ |  | 1.19 (1.06-1.33) | **3.35×10^-3^** |
| **rs7075976** | | 10q11 | A/G |  | 0.409 | 0.336 | 1.35 (1.13-1.61) | 7.81×10^-4^ |  | 0.376 | 0.368 | 1.04 (0.92-1.17) | 5.59×10^-1^ |  | 1.13 (1.02-1.24) | **1.53×10^-2^** |
| **rs2573559** | | 10q22 | A/G |  | 0.426 | 0.501 | 0.74 (0.62-0.88) | 7.38×10^-4^ |  | 0.475 | 0.473 | 1.01 (0.90-1.13) | 8.99×10^-1^ |  | 0.92 (0.84-1.01) | 8.49×10^-2^ |
| **rs7929338** | | 11p15 | T/C |  | 0.099 | 0.155 | 0.59 (0.45-0.77) | 1.51×10^-4^ |  | 0.122 | 0.137 | 0.88 (0.74-1.04) | 1.26×10^-1^ |  | 0.79 (0.68-0.91) | **1.11×10^-3^** |
| **rs4522153** | | 11p15 | T/G |  | 0.187 | 0.248 | 0.70 (0.57-0.86) | 9.87×10^-4^ |  | 0.229 | 0.222 | 1.04 (0.91-1.19) | 6.01×10^-1^ |  | 0.93 (0.83-1.04) | 1.87×10^-1^ |
| **rs12361755** | | 11q13 | G/A |  | 0.396 | 0.468 | 0.74 (0.63-0.89) | 9.40×10^-4^ |  | 0.433 | 0.439 | 0.98 (0.87-1.11) | 7.07×10^-1^ |  | 0.89 (0.81-0.99) | **2.85×10^-2^** |
| **rs6598099** | | 11q13 | C/T |  | 0.278 | 0.212 | 1.44 (1.18-1.77) | 3.97×10^-4^ |  | 0.258 | 0.237 | 1.12 (0.98-1.28) | 8.53×10^-2^ |  | 1.21 (1.08-1.35) | **8.18×10^-4^** |
| **rs7350489** | | 11q21 | C/T |  | 0.055 | 0.098 | 0.55 (0.39-0.77) | 5.53×10^-4^ |  | 0.085 | 0.084 | 1.02 (0.83-1.25) | 8.81×10^-1^ |  | 0.86 (0.72-1.03) | 1.01×10^-1^ |
| **rs2171186** | | 11q21 | T/C |  | 0.357 | 0.290 | 1.38 (1.14-1.66) | 8.29×10^-4^ |  | 0.325 | 0.341 | 0.93 (0.82-1.05) | 2.22×10^-1^ |  | 1.04 (0.94-1.16) | 4.07×10^-1^ |
| **rs10831295** | | 11q21 | A/C |  | 0.335 | 0.269 | 1.40 (1.15-1.69) | 7.51×10^-4^ |  | 0.316 | 0.329 | 0.94 (0.83-1.07) | 3.41×10^-1^ |  | 1.06 (0.95-1.18) | 2.96×10^-1^ |
| **rs11225064** | | 11q22 | G/C |  | 0.242 | 0.308 | 0.71 (0.58-0.86) | 6.97×10^-4^ |  | 0.291 | 0.279 | 1.06 (0.93-1.20) | 3.45×10^-1^ |  | 0.94 (0.85-1.05) | 2.84×10^-1^ |
| **rs2852189** | | 11q22 | C/T |  | 0.150 | 0.208 | 0.68 (0.54-0.85) | 8.26×10^-4^ |  | 0.194 | 0.199 | 0.97 (0.84-1.12) | 6.72×10^-1^ |  | 0.88 (0.78-0.99) | **3.16×10^-2^** |
| **rs3781817** | | 11q23 | C/T |  | 0.385 | 0.309 | 1.39 (1.16-1.67) | 3.45×10^-4^ |  | 0.346 | 0.360 | 0.94 (0.83-1.06) | 3.47×10^-1^ |  | 1.06 (0.96-1.17) | 2.58×10^-1^ |
| **rs10790828** | | 11q24 | T/C |  | 0.432 | 0/504 | 0.74 (0.62-0.88) | 9.10×10^-4^ |  | 0.472 | 0.475 | 0.99 (0.88-1.11) | 8.56×10^-1^ |  | 0.91 (0.82-1.00) | **4.94×10^-2^** |
| **rs3816880** | | 12q13 | C/T |  | 0.097 | 0.059 | 1.79 (1.28-2.51) | 7.21×10^-4^ |  | 0.064 | 0.059 | 1.08 (0.85-1.37) | 5.10×10^-1^ |  | 1.28 (1.05-1.56) | **1.32×10^-2^** |
| **rs2121889** | | 12q21 | T/C |  | 0.406 | 0.331 | 1.35 (1.14-1.61) | 6.75×10^-4^ |  | 0.383 | 0.402 | 0.92 (0.82-1.03) | 1.75×10^-1^ |  | 1.04 (0.94-1.14) | 4.60×10^-1^ |
| **rs9512979** | | 13q12 | A/G |  | 0.043 | 0.016 | 2.70 (1.56-4.68) | 3.96×10^-4^ |  | 0.021 | 0.024 | 0.87 (0.59-1.28) | 4.86×10^-1^ |  | 1.26 (0.92-1.73) | 1.44×10^-1^ |
| **rs9512982** | | 13q12 | G/A |  | 0.042 | 0.016 | 2.63 (1.52-4.56) | 6.07×10^-4^ |  | 0.021 | 0.024 | 0.87 (0.59-1.28) | 4.86×10^-1^ |  | 1.25 (0.91-1.72) | 1.64×10^-1^ |
| **rs9512985** | | 13q12 | C/T |  | 0.042 | 0.016 | 2.63 (1.52-4.56) | 6.07×10^-4^ |  | 0.021 | 0.024 | 0.87 (0.59-1.28) | 4.86×10^-1^ |  | 1.25 (0.91-1.72) | 1.64×10^-1^ |
| **rs9512987** | | 13q12 | G/A |  | 0.042 | 0.016 | 2.63 (1.52-4.56) | 6.07×10^-4^ |  | 0.021 | 0.024 | 0.87 (0.59-1.28) | 4.90×10^-1^ |  | 1.25 (0.91-1.72) | 1.64×10^-1^ |
| **rs1105576** | | 13q32 | C/T |  | 0.453 | 0.380 | 1.36 (1.14-1.62) | 7.80×10^-4^ |  | 0.416 | 0.407 | 1.04 (0.93-1.17) | 5.20×10^-1^ |  | 1.13 (1.02-1.24) | **1.61×10^-2^** |
| **rs881946** | | 13q33 | C/T |  | 0.162 | 0.223 | 0.68 (0.55-0.85) | 5.80×10^-4^ |  | 0.189 | 0.199 | 0.94 (0.81-1.09) | 3.62×10^-1^ |  | 0.85 (0.76-0.96) | **9.42×10^-2^** |
| **rs1330518** | | 13q33 | A/G |  | 0.163 | 0.222 | 0.69 (0.55-0.86) | 8.84×10^-4^ |  | 0.190 | 0.199 | 0.94 (0.81-1.09) | 4.24×10^-1^ |  | 0.86 (0.76-0.97) | **1.12×10^-2^** |
| **rs12873417** | | 13q33 | T/C |  | 0.112 | 0.173 | 0.62 (0.48-0.79) | 1.58×10^-4^ |  | 0.140 | 0.148 | 0.94 (0.80-1.11) | 4.67×10^-1^ |  | 0.83 (0.72-0.95) | **7.25×10^-3^** |
| **rs17769369** | | 14q21 | C/T |  | 0.103 | 0.154 | 0.64 (0.49-0.83) | 8.26×10^-4^ |  | 0.124 | 0.140 | 0.87 (0.73-1.03) | 1.18×10^-1^ |  | 0.79 (0.69-0.92) | **1.54×10^-3^** |
| **rs12593165** | | 15q22 | C/A |  | 0.114 | 0.169 | 0.65 (0.51-0.83) | 6.22×10^-4^ |  | 0.134 | 0.136 | 0.99 (0.84-1.17) | 8.69×10^-1^ |  | 0.87 (0.75-1.00) | **4.22×10^-2^** |
| **rs12101466** | | 15q24 | A/G |  | 0.322 | 0.391 | 0.73 (0.61-0.88) | 9.44×10^-4^ |  | 0.366 | 0.357 | 1.04 (0.92-1.17) | 5.25×10^-1^ |  | 0.94 (0.85-1.04) | 2.10×10^-1^ |
| **rs2012387** | | 15q26 | C/T |  | 0.202 | 0.141 | 1.52 (1.22-1.91) | 2.70×10^-4^ |  | 0.198 | 0.202 | 0.97 (0.84-1.12) | 7.13×10^-1^ |  | 1.10 (0.98-1.24) | 1.09×10^-1^ |
| **rs6498968** | | 16q12 | T/C |  | 0.326 | 0.397 | 0.74 (0.62-0.89) | 9.94×10^-4^ |  | 0.343 | 0.350 | 0.97 (0.86-1.09) | 6.53×10^-1^ |  | 0.89 (0.81-0.99) | **2.38×10^-2^** |
| **rs9900825** | | 17q12 | G/A |  | 0.291 | 0.220 | 1.45 (1.19-1.76) | 2.46×10^-4^ |  | 0.235 | 0.225 | 1.06 (0.92-1.22) | 3.93×10^-1^ |  | 1.17 (1.05-1.31) | **5.27×10^-3^** |
| **rs2008765** | | 17q12 | C/T |  | 0.365 | 0.295 | 1.36 (1.14-1.63) | 9.06×10^-4^ |  | 0.302 | 0.292 | 1.05 (0.93-1.19) | 4.55×10^-1^ |  | 1.14 (1.03-1.26) | **1.17×10^-2^** |
| **rs1474054** | | 17q12 | A/G |  | 0.286 | 0.222 | 1.40 (1.15-1.70) | 9.14×10^-4^ |  | 0.243 | 0.228 | 1.08 (0.94-1.24) | 2.61×10^-1^ |  | 1.18 (1.05-1.32) | **5.00×10^-3^** |
| **rs7239971** | | 18p11 | G/A |  | 0.442 | 0.369 | 1.35 (1.14-1.61) | 7.72×10^-4^ |  | 0.408 | 0.416 | 0.97 (0.86-1.09) | 5.60×10^-1^ |  | 1.07 (0.97-1.18) | 1.51×10^-1^ |
| **rs1893384** | | 18q11 | C/A |  | 0.045 | 0.019 | 2.55 (1.50-4.31) | 5.20×10^-4^ |  | 0.029 | 0.021 | 1.37 (0.94-1.99) | 9.95×10^-2^ |  | 1.69 (1.24-2.29) | **7.71×10^-4^** |
| **rs9956700** | | 18q21 | G/T |  | 0.082 | 0.045 | 1.85 (1.29-2.64) | 8.25×10^-4^ |  | 0.075 | 0.071 | 1.06 (0.85-1.32) | 5.94×10^-1^ |  | 1.24 (1.02-1.49) | **2.75×10^-2^** |
| **rs11659290** | | 18q21 | G/A |  | 0.078 | 0.042 | 1.87 (1.30-2.70) | 7.89×10^-4^ |  | 0.076 | 0.070 | 1.08 (0.87-1.35) | 4.76×10^-1^ |  | 1.25 (1.04-1.51) | **2.00×10^-2^** |
| **rs8091906** | | 18q21 | C/T |  | 0.079 | 0.042 | 1.90 (1.32-2.74) | 5.67×10^-4^ |  | 0.076 | 0.070 | 1.08 (0.87-1.35) | 4.76×10^-1^ |  | 1.26 (1.04-1.52) | **1.76×10^-2^** |
| **rs11662622** | | 18q22 | A/G |  | 0.223 | 0.287 | 0.70 (0.57-0.86) | 6.43×10^-4^ |  | 0.259 | 0.244 | 1.09 (0.96-1.24) | 2.22×10^-1^ |  | 0.96 (0.86-1.07) | 4.46×10^-1^ |
| **rs2143253** | | 20q13 | G/A |  | 0.197 | 0.259 | 0.70 (0.57-0.86) | 8.41×10^-4^ |  | 0.233 | 0.230 | 1.02 (0.89-1.17) | 8.33×10^-1^ |  | 0.91 (0.81-1.02) | 1.13×10^-1^ |
| **rs1557382** | | 21q21 | C/T |  | 0.243 | 0.315 | 0.69 (0.57-0.84) | 2.49×10^-4^ |  | 0.282 | 0.280 | 1.01 (0.89-1.15) | 8.67×10^-1^ |  | 0.90 (0.81-1.00) | 5.77×10^-2^ |
| **rs1788324** | | 21q21 | A/G |  | 0.514 | 0.439 | 1.35 (1.14-1.61) | 6.37×10^-4^ |  | 0.478 | 0.477 | 1.00 (0.89-1.12) | 9.49×10^-1^ |  | 1.10 (1.00-1.21) | 5.87×10^-2^ |
| **rs2838808** | | 21q22 | T/C |  | 0.153 | 0.210 | 0.68 (0.54-0.86) | 9.74×10^-4^ |  | 0.173 | 0.177 | 0.97 (0.83-1.13) | 7.11×10^-1^ |  | 0.87 (0.77-0.99) | **3.04×10^-2^** |
| **rs2241042** | | 22q11 | A/C |  | 0.404 | 0.329 | 1.39 (1.16-1.66) | 3.74×10^-4^ |  | 0.339 | 0.353 | 0.94 (0.83-1.06) | 3.13×10^-1^ |  | 1.06 (0.96-1.17) | 2.53×10^-1^ |
| **rs2241043** | | 22q11 | C/T |  | 0.404 | 0.329 | 1.39 (1.16-1.66) | 3.74×10^-4^ |  | 0.336 | 0.350 | 0.94 (0.83-1.06) | 3.18×10^-1^ |  | 1.06 (0.96-1.17) | 2.47×10^-1^ |
| **rs7289560** | | 22q12 | A/G |  | 0.155 | 0.100 | 1.68 (1.29-2.19) | 1.36×10^-4^ |  | 0.118 | 0.120 | 0.98 (0.82-1.17) | 8.32×10^-1^ |  | 1.16 (1.00-1.34) | 5.31×10^-2^ |
| **rs10483154** | | 22q12 | A/G |  | 0.155 | 0.101 | 1.66 (1.28-2.17) | 1.73×10^-4^ |  | 0.118 | 0.122 | 0.97 (0.81-1.16) | 7.04×10^-1^ |  | 1.14 (0.99-1.32) | 7.20×10^-2^ |
| **rs16987084** | | 22q12 | T/C |  | 0.155 | 0.103 | 1.61 (1.24-2.10) | 3.72×10^-4^ |  | 0.118 | 0.122 | 0.96 (0.81-1.14) | 6.76×10^-1^ |  | 1.13 (0.97-1.30) | 1.07×10^-1^ |
| **rs8135301** | | 22q12 | G/T |  | 0.154 | 0.103 | 1.60 (1.23-2.08) | 4.87×10^-4^ |  | 0.118 | 0.122 | 0.96 (0.81-1.14) | 6.76×10^-1^ |  | 1.12 (0.97-1.30) | 1.17×10^-1^ |
| **rs132304** | | 22q12 | A/G |  | 0.133 | 0.087 | 1.61 (1.22-2.13) | 7.94×10^-4^ |  | 0.116 | 0.107 | 1.09 (0.91-1.31) | 3.31×10^-1^ |  | 1.23 (1.05-1.43) | **8.76×10^-3^** |
| **rs4415** | | 22q13 | G/A |  | 0.347 | 0.270 | 1.44 (1.19-1.74) | 1.47×10^-4^ |  | 0.328 | 0.321 | 1.03 (0.91-1.16) | 6.06×10^-1^ |  | 1.14 (1.03-1.26) | **1.34×10^-2^** |
| **rs35662666** | | 22q13 | C/T |  | 0.241 | 0.179 | 1.47 (1.19-1.83) | 4.63×10^-4^ |  | 0.220 | 0.209 | 1.07 (0.93-1.23) | 3.61×10^-1^ |  | 1.18 (1.04-1.32) | **6.84×10^-3^** |

Abbreviations: GWAS, genome-wide association study; OR, odds ratio; CI, confidence intervals, *P*_het_, *P* value for Cochran’s Q statistic for between-study heterogeneity; *I*^2^, index of heterogeneity.

^a^Major/minor allele.

^b^Previously reported prostate cancer-associated SNPs.

NOTE: SNPs with significant association with prostate cancer (*P*<0.05) are in bold and those with *P*<1×10^-4^ are highlighted in green in corresponding columns, except for results obtained in iCOGS array. Horizontal dashed lines separate SNPs in LD with reported prostate cancer-associated SNP.
